# Alpelisib combination treatment as novel targeted therapy against hepatocellular carcinoma

**DOI:** 10.1038/s41419-021-04206-5

**Published:** 2021-10-08

**Authors:** Hongwei Xu, Kefei Chen, Runze Shang, Xinyan Chen, Yi Zhang, Xinhua Song, Matthias Evert, Sheng Zhong, Bo Li, Diego F. Calvisi, Xin Chen

**Affiliations:** 1 Department of Liver Surgery, Center of Liver Transplantation, West China Hospital of Sichuan University, Chengdu, Sichuan China; 2 Department of Bioengineering and Therapeutic Sciences and Liver Center, University of California, San Francisco, CA USA; 3 Department of General Surgery, Affiliated Haixia Hospital of Huaqiao University, The 910 Hospital, Quanzhou, Fujian China; 4 Department of Pharmacy, Hubei University of Chinese Medicine, Wuhan, Hubei China; 5 Key Laboratory of Biorheological Science and Technology, Ministry of Education, College of Bioengineering, Chongqing University, Chongqing, China; 6 Institute of Pathology, University of Regensburg, Regensburg, Germany

**Keywords:** Drug development, Drug development

## Abstract

Hepatocellular carcinoma (HCC) is the sixth most common primary cancer with an unsatisfactory long-term survival. Gain of function mutations of *PIK3CA* occur in a subset of human HCC. Alpelisib, a selective PIK3CA inhibitor, has been approved by the FDA to treat *PIK3CA* mutant breast cancers. In this manuscript, we evaluated the therapeutic efficacy of alpelisib, either alone or in combination, for the treatment of HCC. We tested alpelisib in mouse HCC induced by hydrodynamic injection of c-Met/PIK3CA(H1047R) (c-Met/H1047R), c-Met/PIK3CA(E545K) (c-Met/E545K), and c-Met/sgPten gene combinations. Alpelisib slowed down the growth of c-Met/H1047R and c-Met/E545K HCC but was ineffective in c-Met/sgPten HCC. Mechanistically, alpelisib inhibited p-ERK and p-AKT in c-Met/H1047R and c-Met/E545K HCC progression but did not affect the mTOR pathway or genes involved in cell proliferation. In human HCC cell lines transfected with PIK3CA(H1047R), alpelisib synergized with the mTOR inhibitor MLN0128 or the CDK4/6 inhibitor palbociclib to suppress HCC cell growth. In c-Met/H1047R mice, alpelisib/MLN0128 or alpelisib/palbociclib combination therapy caused tumor regression. Our study demonstrates that alpelisib is effective for treating *PIK3CA-*mutated HCC by inhibiting MAPK and AKT cascades. Furthermore, combining alpelisib with mTOR or CDK4/6 inhibitors has a synergistic efficacy against *PIK3CA*-mutated HCC, providing novel opportunities for precision medicine against HCC.

## Introduction

Hepatocellular carcinoma (HCC) is currently ranked as the sixth most common primary cancer and the fourth leading cause of cancer-related deaths worldwide [1]. Sorafenib, a multi-kinase inhibitor, has been the first-line treatment drug for HCC in the past decade, but it has limited efficacy [2]. Recently, additional multi-kinase inhibitors, including cabozantinib and regorafenib, have been approved for HCC treatment [3]. Importantly, immunotherapy has demonstrated significant therapeutic efficacy against advanced HCC, and it has now become the first-line treatment option for advanced HCC [4]. However, most patients eventually progress with all these therapies. Despite all this progress, tumor genetics-specific-targeted therapies against HCC are unsatisfactory. Therefore, the development of novel treatment strategies against HCC, especially biomarker-based targeted therapies, is imperative.

Recent advances in whole-genome sequencing of HCC have provided a more comprehensive understanding of this heterogeneous malignancy [5, 6]. Multiple mutations in various genes, such as *TP53*, *CTNNB1*, *AXIN1*, *ARID1A/*B, and *ARID2*, have been detected in human HCC subsets [5]. Among them, gain of function (GOF) mutations of *PIK3CA* account for ~3% and rank among the top 20 mutations in human HCC based on the COSMIC database (Supplementary Fig. S1). Despite the relatively low percentage of patients with GOF PIK3CA mutations, since ~850,000 cases of HCC are diagnosed per year, ~25,000 HCC patients carry this specific genetic event [7]. In the era of precision medicine, these patients could be readily identified and may benefit from anti-PIK3CA based therapies.

*PIK3CA* encodes the phosphatidylinositol 3-kinase (PI3K) p110α, which is critical in cancer development [8, 9]. In physiological conditions, PI3Ks are activated by receptor tyrosine kinases and G-protein coupled receptors. Following activation, PI3Ks phosphorylate the lipid substrate phosphatidylinositol (4,5)-bisphosphate into phosphatidylinositol (3,4,5)-trisphosphate (PIP3). PIP3 acts as a second messenger that amplifies the PI3K/AKT/mTOR signaling in regulating cell proliferation, survival, metabolism, and motility [10]. GOF *PIK3CA* mutations deregulate the PI3K/AKT/mTOR cascade and ultimately lead to cancer formation. PIK3CA mutations map to three sites, E542 and E545, at the helical domain (exon 9; E542K/E545K), and H1047, in the kinase domain (exon 20; H1047R) [9]. In murine HCCs, previous studies from our lab showed that overexpression of H1047R or E545K together with c-Met or NRasV12 could induce liver tumor formation in mice, providing preclinical models to explore HCCs with GOF *PIK3CA* mutations [11].

Alpelisib, an orally administered PIK3CA inhibitor, has been approved by the FDA to treat breast cancers with *PIK3CA* mutations. Both preclinical and clinical studies have reported significant efficiency of alpelisib against tumors with GOF *PIK3CA* mutations [12–16]. However, whether alpelisib could function to suppress the growth of *PIK3CA*-mutated HCC has never been investigated. In the present study, we explored the therapeutic efficacy of alpelisib, either alone or in combination with other drugs, such as the mTOR inhibitor MLN0128 or the CDK4/6 inhibitor palbociclib against GOF *PIK3CA*-mutated HCC both in vitro and in vivo.

## Results

### *PIK3CA* mutation status in human HCC samples

To characterize the molecular functions of *PIK3CA* in liver tumors, we analyzed the *PIK3CA* genomic alterations in human HCCs based on the COSMIC database [17]. We found that *PIK3CA* mutations occur in 0.9–3.6% HCC samples (Supplementary Fig. S1a). Among the 97 mutations, over 70% consist of GOF missense substitutions (Supplementary Fig. S1b). These data indicate that *PIK3CA* mutations could be identified in ~3% human HCCs; thus, anti-PIK3CA therapy may represent a promising approach against this HCC subset.

### *PIK3CA* mutations increase HCC cell sensitivity to alpelisib in vitro

First, we treated eight human HCC cell lines, none of them harboring GOF *PIK3CA* mutations, with alpelisib. In these cell lines, alpelisib inhibited cell growth with various IC_50_ values, ranging from 25 to 160 μM (Supplementary Fig. S2), supporting the general toxicity of the PIK3CA inhibitor towards HCC cell lines.

**Fig. 1:**
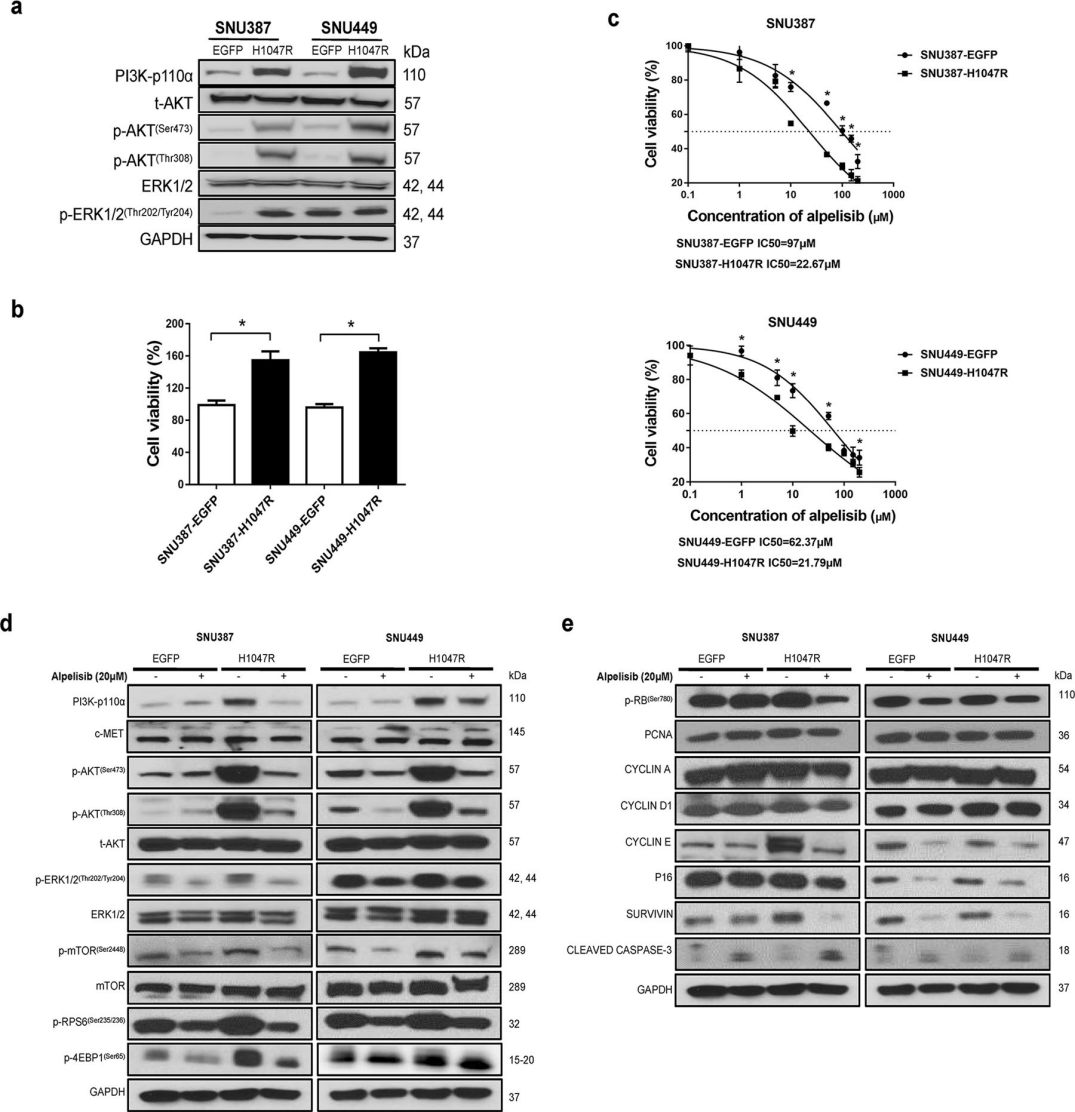
Transfection of PIK3CAH1047R mutation increases the sensitivity to alpelisib treatment of HCC cells. **a** Transfection of PIK3CAH1047R mutation construct by lentivirus and activation of its downstream effectors in SNU387 and SNU449 cell lines was confirmed by western blot analysis. **b** Transfected cell lines were seeded in 24-well plates at 2.5 × 105 for 48 h, and cell viability was calculated. **c** Transfected cell lines were treated with escalating concentrations of alpelisib for 48 h, and IC50 values were calculated. **d** Western blot analysis of AKT/mTOR and Ras/MAPK pathways after treatment with alpelisib. **e** Western blot analysis of cell-cycle-related proteins after treatment with alpelisib. Statistical significance of at least *P* < 0.05 is denoted by asterisk (*).

Next, we hypothesized that alpelisib might be more effective against HCC with GOF *PIK3CA* mutations. Thus, two HCC cell lines with relatively low expression of PI3K and high expression of c-MET, namely SNU449 and SNU387 cells (Supplementary Fig. S3), were transfected with *PIK3CA* mutants, *PIK3CAH1047R* (H1047R) and *PIK3CAE545K* (E545K), to test this hypothesis. Successful transfection of H1047R was confirmed by western blot analysis, showing an increased expression of PI3K-p110α level (Fig. 1a). Overexpression of H1047R led to augmented HCC proliferation and decreased IC_50_ values against alpelisib, consistent with the increased sensitivity to alpelisib (Fig. 1b and c). At the molecular level, alpelisib treatment inhibited the PI3K/AKT/mTOR pathway by suppressing the levels of p-AKT, p-mTOR, p-RPS6, and p-4EBP1. Also, alpelisib effectively downregulated the Ras/MAPK and cell proliferation pathways, as indicated by decreased p-ERK and p-RB expression (Fig. 1d and e; Supplementary Fig. S4).

Next, we transfected SNU449 and SNU387 cells with *PIK3CAE545K* (E545K). Overexpression of E545K also increased the sensitivity of HCC cells to alpelisib, although the increased sensitivity was much milder than that observed in H1047R transfected cells (Supplementary Fig. S5). The molecular targets of alpelisib in E545K transfected cells were the same unraveled in H1047R overexpressing cells (Supplementary Figs. S6 and S7).

Overall, the data indicate that the overexpression of *PIK3CA* mutants increases the sensitivity of HCC cell lines to alpelisib treatment.

### Alpelisib treatment decreases tumor burden in c-Met/H1047R and c-Met/E545K murine HCC models

Next, we assessed the therapeutic efficacy of alpelisib in vivo, using murine HCC models. Previously, we have shown that overexpression of PIK3CAH1047R or PIK3CAE545K alone failed to induce HCC formation in mice. Yet, these genes cooperated with overexpression of c-Met to drive hepatocarcinogenesis (c-Met/H1047R and c-Met/E545K mice) [11].

**Fig. 2:**
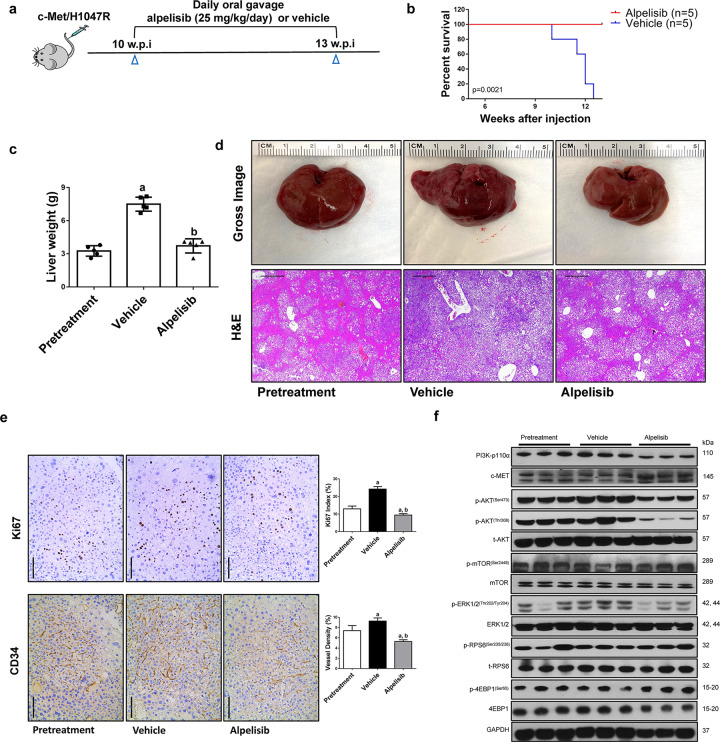
Alpelisib treatment induces stable disease in c-Met/H1047R mice. **a** Study design. w.p.i., weeks post-injection. **b** Survival curve of c-Met/H1047R mice pretreated, treated with alpelisib, and vehicle. **c** Liver weight of pretreatment, vehicle-, and alpelisib-treated c-Met/H1047R mice. **d** Gross images and H&E staining of livers from pretreatment, vehicle-, and alpelisib-treated c-Met/H1047R mice. Magnification ×40; scale bar = 500 μm. **e** Ki67 (magnification ×200; scale bar = 100 μm) and CD34 (magnification ×200; scale bar = 100 μm) staining in livers from c-Met/H1047R mice. Ki67-positive cells were counted and quantified as a proliferation index. CD34 staining was quantified and represented as the percentage of the positive staining area of the whole section area. Tukey–Kramer test: at least *P* < 0.05. **a** vs. Pretreatment; **b** vs. Vehicle. **f** Western blot analysis was performed to determine the level of AKT/mTOR and Ras/MAPK pathways in pretreatment, vehicle-, and alpelisib-treated c-Met/H1047R mice.

First, we tested alpelisib in the c-Met/H1047R model. As we and others previously published, total liver weight was used to estimate liver tumor burden [18–21]. In brief, mice were injected with c-Met/H1047R; 10 weeks post-injection, a group of mice was harvested as the pretreatment cohort, with the mice showing moderate liver tumor burden, i.e., liver weight ~3 g. Additional cohorts of mice were treated with vehicle or alpelisib, starting the administration at 10 weeks post-injection (Fig. 2a). These mice were harvested when they developed large abdominal masses or after 3 weeks of treatment. We found that all mice in the vehicle group developed large tumor masses and required euthanasia. In contrast, alpelisib-treated mice were alive and in relatively good conditions when harvested after 3 weeks of treatment (Fig. 2b). Notably, liver weight in the alpelisib cohort was lower than that from the vehicle cohort and similar to that of pretreatment tumor burden, indicating that alpelisib treatment led to stable disease in c-Met/H1047R mice (Fig. 2c).

We repeated alpelisib treatment studies using the c-Met/E545K HCC model (Supplementary Fig. S8a). Consistent with the in vitro studies indicating that alpelisib is less effective against E545K mutations, alpelisib treatment did not improve the overall survival rate (Supplementary Fig. S8b). Nevertheless, alpelisib treatment decreased overall tumor burden compared with vehicle-treated mice, indicating alpelisib retarded c-Met/E545K HCC progression (Supplementary Fig. S8c).

At the histological level, alpelisib treatment did not affect the histopathological patterns of c-Met/H1047R HCC lesions. All three groups of mice showed similar neoplastic lesions across the whole liver, characterized by clear cells with enlarged cytoplasm (Fig. 2d). Using the percentage of Ki67(+) cells as the proliferation index, alpelisib inhibited HCC cell proliferation (Fig. 2e). Few apoptotic cells were detected within the tumor lesions, and alpelisib did not induce additional HCC apoptosis (Supplementary Fig. S9). A robust decline of vessel density was observed in alpelisib-treated mice (Fig. 2e) when examining tumor vascularity. At the molecular level, alpelisib treatment significantly decreased the expression of p-ERK1/2, p-AKT^(Ser473)^, and p-AKT^(Thr308)^ (Fig. 2f; Supplementary Fig. S10). However, unlike the in vitro data (Fig. 1d), alpelisib failed to suppress the AKT/mTOR pathway downstream effectors, as p-RPS6 and p-4EBP1 levels remained unchanged. These findings were validated by IHC (Fig. 2f; Supplementary Figs. S10 and S11). Moreover, alpelisib inhibited the expression of p-STAT3^(Tyr705)^ and YAP in tumor lesions (Supplementary Fig. S12a). Even though alpelisib-treated HCC exhibited decreased cell proliferation, the primary regulators of cell proliferation were not affected by alpelisib administration (Supplementary Figs. S10 and S13).

In the c-Met/E545K HCC model, alpelisib did not decrease the Ki67 index or increase apoptosis but suppressed angiogenesis, leading to decreased vascular density (Supplementary Figs. S14, S15, and S16). Alpelisib reduced p-ERK1/2 and p-AKT^(Ser473)^ levels, although the inhibition was milder than observed in the c-Met/H1047R model (Supplementary Figs. S17 and S18). Consistent with c-Met/H1047R HCC, alpelisib could not restrain the mTOR pathway (Supplementary Figs. S17 and S18). Unlike c-Met/H1047R HCC, alpelisib did not suppress p-STAT3^(Tyr705)^ and YAP expression in c-Met/E545K HCC (Supplementary Fig. S12B).

The data show that alpelisib treatment induces stable disease in c-Met/H1047R mouse HCC via inhibition of MAPK and AKT pathways. Alpelisib only slows down c-Met/E545K HCC growth.

### Alpelisib is ineffective against c-Met/sgPten-dependent hepatocarcinogenesis

To evaluate whether alpelisib is specifically effective against *PIK3CA*-mutated HCC, we generated the c-Met/sgPten mouse model via the overexpression of c-Met and loss of Pten [22]. The mice were aged until 9 weeks post-injection before intervention (Supplementary Fig. S19a). Similar survival rates and tumor burden were detected in vehicle- and alpelisib-treated mice (Supplementary Fig. S19b and c). Immunostaining confirmed the deletion of PTEN and alpelisib treatment did not decrease tumor proliferation (Supplementary Fig. S20). Consistently, no significant downregulation of MAPK/ERK-, AKT/mTOR-, or cell proliferation-related proteins was detected (Supplementary Figs. S21 and S22). Thus, the data indicate that alpelisib has no therapeutic efficacy against c-Met/sgPten murine HCC, supporting the specificity of alpelisib against *PIK3CA*-mutated HCC.

### Alpelisib-based combination treatment in vitro

**Fig. 3:**
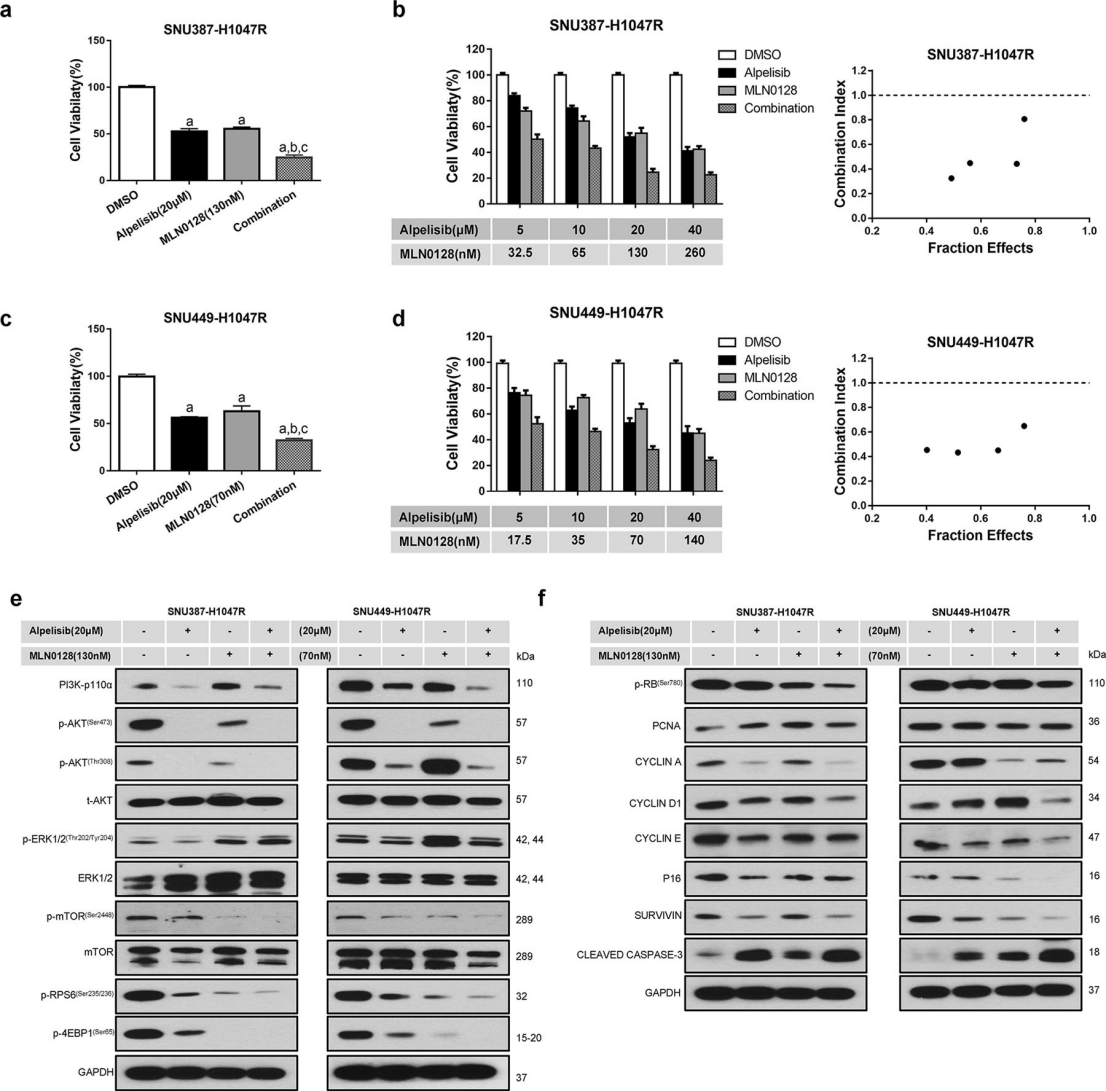
Effects of combined alpelisib/MLN0128 treatment on SNU387-H1047R and SNU449-H1047R cell lines. Transfected cell lines were seeded in 24-well plates at 2.5 × 10^5^ and treated for 48 h. **a**, **c** Combined alpelisib**/**MLN0128 treatment reduced cell proliferation in SNU387-H1047R and SNU449-H1047R cells. **b**, **d** The enhanced inhibitory effect of the alpelisib/MLN0128 combination on HCC in vitro growth is a synergistic action. **e** Western blot analysis of AKT/mTOR and Ras/MAPK pathways in SNU387-H1047R and SNU449-H1047R cell lines. **f** Western blot analysis of proliferation signaling pathways in SNU387-H1047R and SNU449-H1047R cell lines. Tukey–Kramer test: at least *P* < 0.05. **a** vs. DMSO; **b** vs. Alpelisib; **c** vs. MLN0128.

**Fig. 4:**
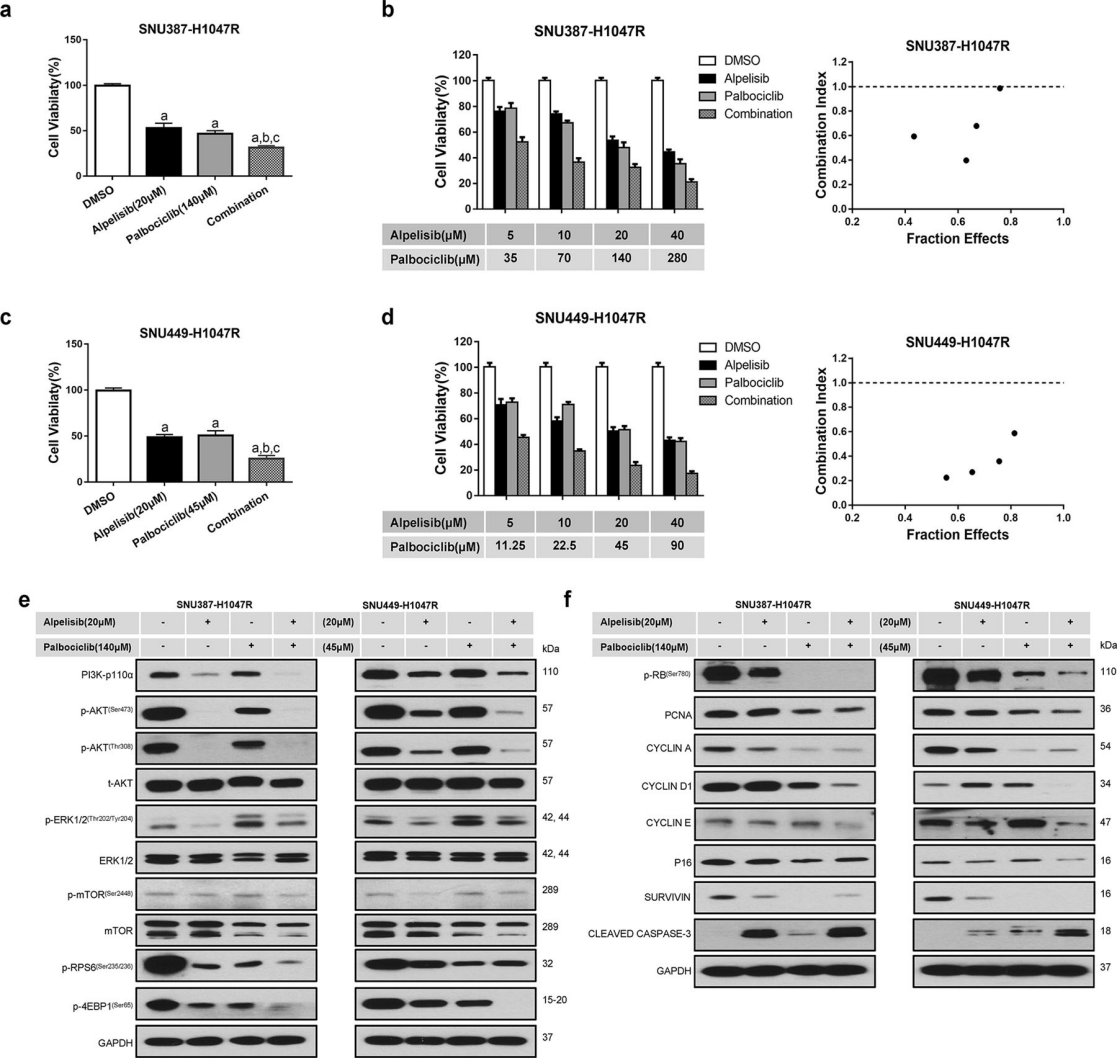
Effects of combined alpelisib/palbociclib treatment on SNU387-H1047R and SNU449-H1047R cell lines. Transfected cell lines were seeded in 24-well plates at 2.5 × 10^5^ and treated for 48 h. **a,** **c** Combined alpelisib/p**a**lboci**c**lib treatment reduced cell proliferation in SNU387-H1047R and SNU449-H1047R cells. **b**, **d** The enhanced inhibitory effect of the alpelisib/palbociclib combination on HCC in vitro growth is a synergistic action. **e** Western blot analysis of AKT**/**mTOR and Ras/MAPK pathways in SNU387-H1047R and SNU449-H1047R cell lines. **f** Western blot analysis of proliferation signaling pathways in SNU387-H1047R and SNU449-H1047R cell lines. Tukey–Kramer test: at least *P* < 0.05. **a** vs. DMSO; **b** vs. Alpelisib; **c** vs. Palbociclib.

The above studies indicate that alpelisib monotherapy may have mild to moderate therapeutic efficacy against *PIK3CA*-mutated HCC and suggest the requirement of combination therapy for effective treatment. Using SNU387-H1047R and SNU449-H1047R cell lines, we discovered three drugs that synergized with alpelisib to suppress cell growth: the pan-mTOR inhibitor MLN0128 (Fig. 3), the CDK4/6 inhibitor palbociclib (Fig. 4), and the multi-kinase inhibitor cabozantinib (Supplementary Figs. S23 and S24).

For MLN0128, combined alpelisib and MLN0128 administration resulted in a more potent inhibition activity of HCC cell growth than alpelisib or MLN0128 alone (Fig. 3a and c). The doses used in combination studies were based on the alpelisib and MLN0128’s IC_50_s in SNU387 and SNU449 cell lines (Supplementary Fig. S25). We calculated the combination index (CI), and all combination regimens showed less than 1 CI value, suggesting a synergistic antitumor effect (Fig. 3b and d). MLN0128 treatment alone significantly suppressed the AKT/mTOR cascade. This effect was pronouncedly augmented by the combination of alpelisib, as indicated by the further decline of p-AKT, p-mTOR, p-RPS6, and p-4EBP1 expression upon combination (Fig. 3e; Supplementary Fig. S26). However, compared with alpelisib treatment, MLN0128 administration triggered the upregulation of p-ERK1/2 levels, and the combinatory treatment did not decrease p-ERK1/2 expression. Noticeably, a significant decline of p-RB and CYCLIN D1 levels was observed following the combination treatment, indicating a synergistic antiproliferation effect (Fig. 3f; Supplementary Fig. S26).

Combining alpelisib and palbociclib also resulted in synergistic effects (Fig. 4a-d; Supplementary Fig. S25). Indeed, the alpelisib and palbociclib combination demonstrated enhanced inhibition of the AKT/mTOR pathway (Fig. 4e; Supplementary Fig. S27). Alpelisib inhibited, but palbociclib enhanced p-ERK1/2 levels. Alpelisib blunted the elevated p-ERK1/2 levels induced by palbociclib. Significantly, cell cycle regulators including p-RB, PCNA, CYCLIN A, CYCLIN D1, and CYCLIN E were profoundly downregulated by combining alpelisib and palbociclib (Fig. 4f; Supplementary Fig. S27), supporting the synergistic effects in inhibiting HCC cell proliferation.

The combination of alpelisib and cabozantinib provided similar results, showing synergistic inhibition of HCC growth (Supplementary Fig. S23). Combined alpelisib/cabozantinib treatment displayed further suppression of the AKT/mTOR pathway, as indicated by the downregulation of p-AKT, p-RPS6, and p-4EBP1 levels. In addition, both drugs demonstrated potent inhibition of the p-ERK1/2 levels. The drug combination did not result in further inhibition of p-ERK1/2 (Supplementary Fig. S24).

### Combined alpelisib/MLN0128 treatment leads to tumor regression in c-Met/H1047R mice

**Fig. 5:**
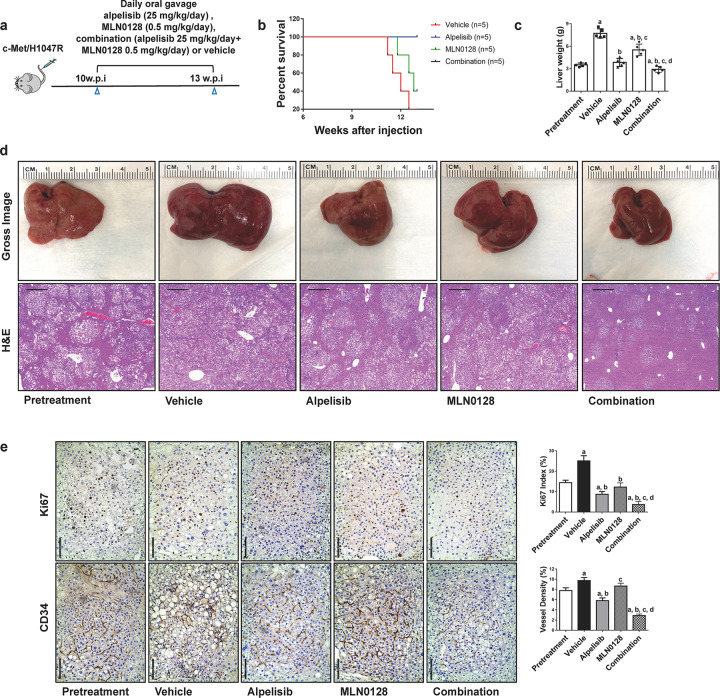
Combined alpelisib/MLN0128 induces tumor regression in HCC lesions from c-Met/H1047R mice. **a** Study design. w.p.i., weeks post-injection. **b** Survival curve of c-Met/H1047R mice pretreated, treated with vehicle, alpelisib, MLN0128, and alpelisib/MLN0128. **c** Liver weight of pretreated, treated with vehicle, alpelisib, MLN0128, and alpelisib/MLN0128 c-Met/H1047R mice. **d** Gross images and H&E staining of livers from pretreated, treated with vehicle, alpelisib, MLN0128, and alpelisib/MLN0128 c-Met/H1047R mice. Magnification ×40; scale bar = 500 μm. **e** Ki67 (magnification ×200; scale bar = 100 μm) and CD34 (magnification ×200; scale bar = 100 μm) staining in livers from c-Met/H1047R mice. Ki67-positive cells were counted and quantified as a proliferation index. CD34 staining was quantified and represented as the percentage of the positive staining area of the whole section area. Tukey–Kramer test: at least *P* < 0.05. **a** vs. Pretreatment; **b** vs. vehicle; **c** vs. Alpelisib; **d** vs. MLN0128.

**Fig. 6:**
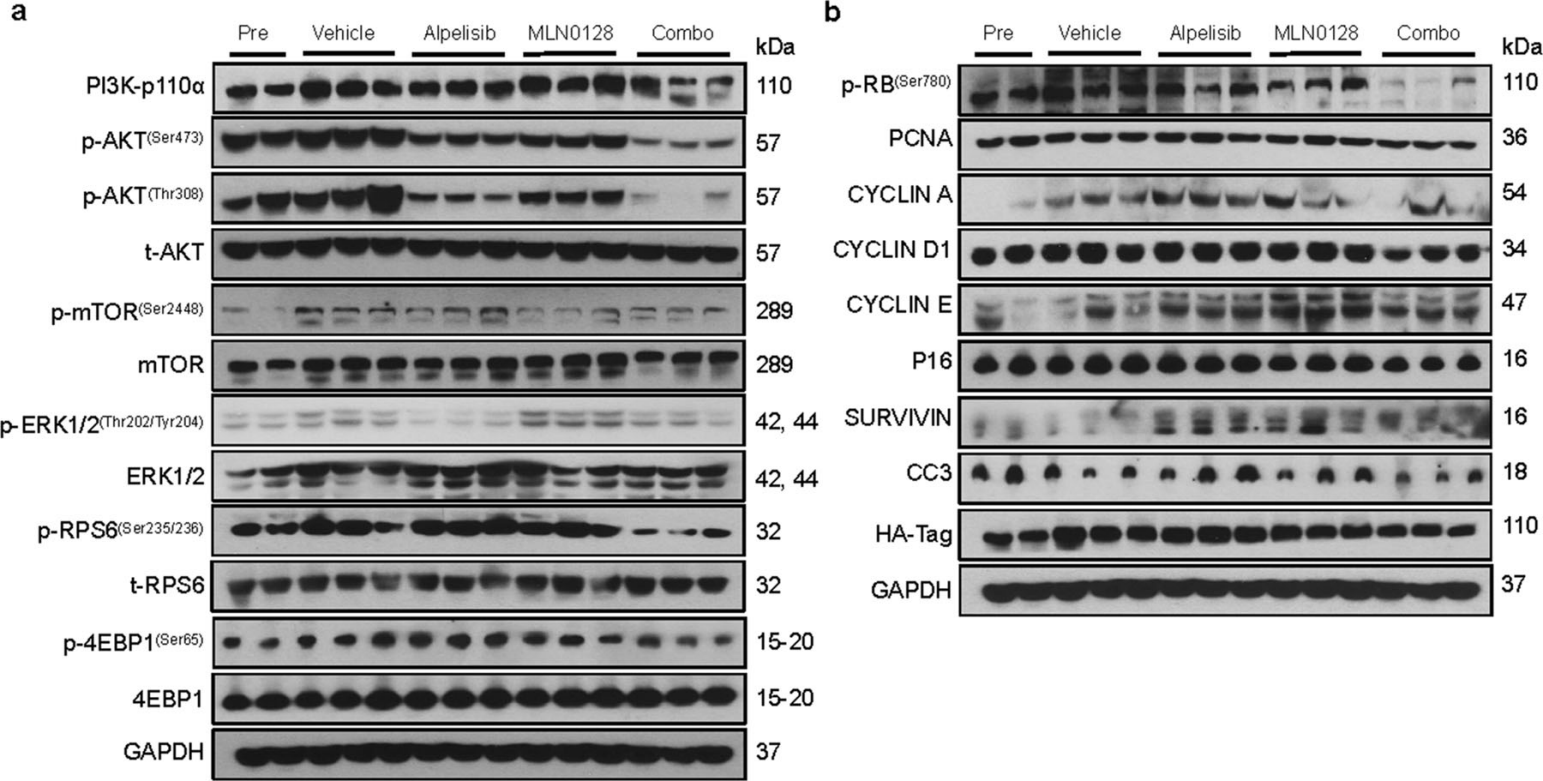
Effects of alpelisib/MLN0128 treatment on the levels of putative target proteins in livers from c-Met/H1047R mice. **a** Western blot analysis of AKT/mTOR and Ras/MAPK pathways in pretreated, treated with vehicle, alpelisib, MLN0128, and alpelisib/MLN0128 c-Met/H1047R mice. **b** Western blot analysis of proliferation signaling pathways in pretreated, treated with vehicle, alpelisib, MLN0128, and alpelisib/MLN0128 c-Met/H1047R mice. Pre Pretreatment, Combo combined alpelisib/MLN0128 treatment, CC3 CLEAVED CASPASE-3.

To further evaluate the antitumor effect of alpelisib/MLN0128 treatment, we applied this combination regimen in c-Met/H1047R mice. Ten weeks after injection, mice were divided into five groups. Group 1 was harvested as a pretreatment group. The remaining mice were subsequently treated and referred to as vehicle, alpelisib, MLN0128, and alpelisib/MLN0128, respectively (Fig. 5a). During the following 3 weeks of observation, all mice in the vehicle group and half of the mice in the MLN0128 group developed lethal tumor burdens and required euthanasia. In contrast, mice in alpelisib and combination groups were in relatively good health by the end of the treatment period (Fig. 5b). MLN0128 led to tumor progression but at a slower rate compared with the vehicle group. Strikingly, alpelisib/MLN0128 treatment led to a lower tumor burden than the pretreatment cohort, suggesting tumor regression upon the combination (Fig. 5c). At the histopathological level, tumor lesions in all five groups displayed a similar phenotype (Fig. 5d). A drastic decline of proliferation and decreased tumor vascular density were observed in the alpelisib/MLN0128 group (Fig. 5e). In addition, the AKT/mTOR pathway was profoundly inhibited when mice were treated with alpelisib/MLN0128, as evidenced by the downregulation of the p-AKT, p-mTOR, p-RPS6, and p-4EBP1 compared with the vehicle or monotherapy groups (Fig. 6a; Supplementary Fig. S28). MLN0128 treatment led to the increased p-ERK expression, which was significantly attenuated in the combination treatment. Consistent with the in vitro data, a significant decline of the p-RB levels was also detected in the combination group (Fig. 6b; Supplementary Fig. S28).

In summary, these data indicate that the combination of alpelisib and MLN0128 induces tumor regression in the c-Met/H1047R mouse HCC model via suppressing MAPK, AKT/mTOR, and cell proliferation pathways.

### Combined alpelisib/palbociclib treatment leads to tumor regression in c-Met/H1047R mice

**Fig. 7:**
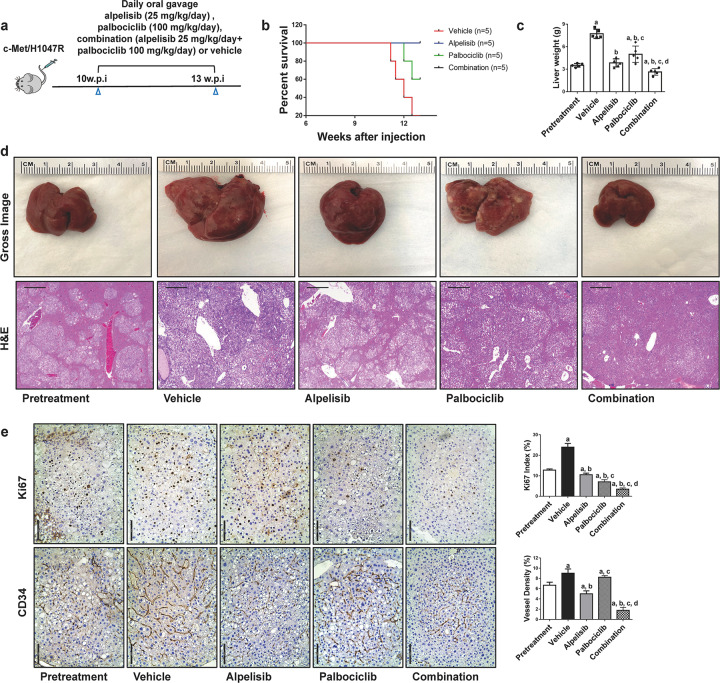
Combined alpelisib/palbociclib induces tumor regression in HCC lesions from c-Met/H1047R mice. **a** Study design. w.p.i., weeks post-injection. **b** Survival curve of c-Met/H1047R mice pretreated, treated with vehicle, alpelisib, palbociclib, and alpelisib/palbociclib. **c** Liver weight of pretreated, treated with vehicle, alpelisib, palbociclib, and alpelisib/palbociclib c-Met/H1047R mice. **d** Gross images and H&E staining of livers from pretreated, treated with vehicle, alpelisib, palbociclib, and alpelisib/palbociclib c-Met/H1047R mice. Magnification ×40; scale bar = 500 μm. **e** Ki67 (magnification ×200; scale bar = 100 μm) and CD34 (magnification ×200; scale bar = 100 μm) staining in livers from c-Met/H1047R mice. Ki67-positive cells were counted and quantified as a proliferation index. CD34 staining was quantified and represented as the percentage of the positive staining area of the whole section area. Tukey–Kramer test: at least *P* < 0.05. **a** vs. Pretreatment; **b** vs. vehicle; **c** vs. Alpelisib; **d** vs. Palbociclib.

**Fig. 8:**
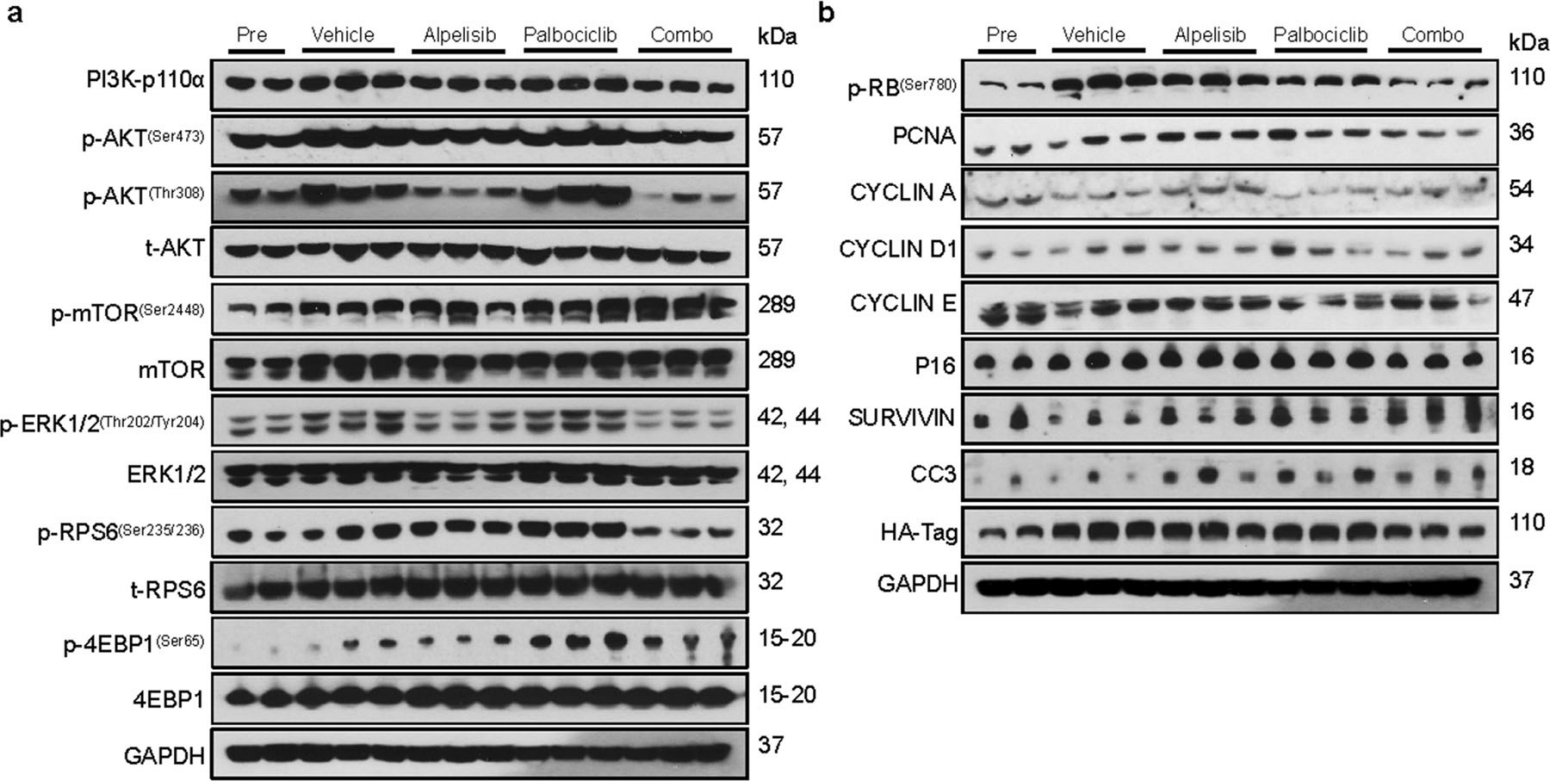
Effects of alpelisib/palbociclib treatment on the levels of putative target proteins in livers from c-Met/H1047R mice. **a** Western blot an**a**lysis of AKT/mTOR and Ras/MAPK pathways in pretreated, treated with vehicle, alpelisib, palbociclib, and alpelisib/palbociclib c-Met/H1047R mice. **b** Western blot analysis of proliferation signaling pathways in pretreated, treated with vehicle, alpelisib, palbociclib, and alpelisib/palbociclib c-Met/H1047R mice. Pre Pretreatment, Combo combined alpelisib/palbociclib treatment, CC3 CLEAVED CASPASE-3.

Next, we evaluated alpelisib/palbociclib combination therapy in the c-Met/H1047R model. Mice were divided into similar five groups (Fig. 7a). During the treatment period, mice in vehicle and palbociclib groups developed relatively larger tumor burdens and needed euthanasia. In contrast, mice treated with alpelisib and the combination regimen exhibited good health (Fig. 7b). The palbociclib group showed the most considerable tumor burden compared with the pretreatment group, indicating the progression of HCC despite the palbociclib administration. Remarkably, the combination group showed a lower tumor burden than pretreatment mice, suggesting tumor regression upon alpelisib/palbociclib treatment (Fig. 7c and d). Furthermore, Alpelisib/palbociclib combination significantly decreased cell proliferation and vascular density (Fig. 7e). At the molecular level, the combination therapy effectively inhibited the mTOR pathway, as indicated by the significant decrease of p-RPS6 (Fig. 8a; Supplementary Fig. S29). In addition, consistent with the remarkably declined proliferation index, alpelisib/palbociclib treatment led to a strong downregulation of p-RB, PCNA, and CYCLIN A levels (Fig. 8b; Supplementary Fig. S29).

Altogether, these data indicate that the alpelisib and palbociclib combination induces tumor regression in c-Met/H1047R mice by suppressing MAPK, AKT/mTOR, and cell proliferation pathways.

### Combined alpelisib/cabozantinib treatment does not increase the therapeutic efficacy in c-Met/H1047R mice

Finally, we investigated the combination of alpelisib/cabozantinib in vivo. The overall study design was similar to the previously described (Supplementary Fig. S30a). We found that cabozantinib monotherapy slowed tumor progression. Intriguingly, in contrast to the in vitro studies, alpelisib/cabozantinib combination therapy did not increase efficacy, as revealed by liver tumor burden and overall survival rate (Supplementary Fig. S30b and c). Alpelisib or cabozantinib alone suppressed the anti-CD34 and Ki67 positivity, but their combination did not lead to an increased inhibitory effect (Supplementary Fig. S31). Mechanistically, cabozantinib monotherapy decreased the levels of p-MET and p-ERK1/2, and alpelisib suppressed p-AKT and p-ERK1/2 expression. However, no synergistic downregulation of either the MAPK pathway or the Akt/mTOR cascade occurred upon the alpelisib/cabozantinib combination (Supplementary Fig. S32).

In conclusion, these findings demonstrate that combined alpelisib/cabozantinib treatment does not increase the therapeutic efficacy in c-Met/H1047R mice.

## Discussion

HCC is a highly heterogeneous malignancy with multiple oncogenes contributing to tumor initiation and progression [5]. Current drugs in clinical practice, such as sorafenib and regorafenib, provide a survival benefit to a relatively small subset of HCC patients [2, 23]. Immunotherapy is a novel and effective therapy against HCC. However, most HCC patients still progress under these regimens. The concept of precision medicine suggests that the treatment for each patient should be tailored against the specific genetic background of the tumors. Unfortunately, currently, no biomarker-based-targeted therapy for HCC treatment exists. Genetic studies have uncovered the mutational landscape of HCC and revealed several possible actionable mutations. One of such alterations is the GOF *PIK3CA* mutation. In the present study, we specifically investigated the therapeutic potency of alpelisib in *PIK3CA*-mutated HCCs, either alone or in combination, using both in vitro and in vivo models. To the best of our knowledge, this is the first study showing a therapeutic effect of alpelisib in *PIK3CA*-mutated HCC. Our investigation demonstrates that alpelisib monotherapy has mild to moderate efficacy against *PIK3CA*-mutated HCC. Importantly, alpelisib synergizes with pan-mTOR or CDK4/6 inhibitors to induce HCC regression.

One intriguing observation of the present data is that H1047R, the kinase domain *PIK3CA* mutation, is more sensitive to alpelisib than E545K, the helical domain *PIK3CA* mutation. This is reflected by the fact that in cell lines, IC_50_ values of alpelisib are ~20 µM against H1047R overexpressing cells, whereas the IC_50_ values are ~45 µM against E545K overexpressing cells. In mouse HCC models, alpelisib treatment induced robust cell proliferation inhibition, leading to stable disease in H1047R/c-Met mice (Fig. 2). However, alpelisib only led to slower tumor progression in E545K/c-Met mice (Supplementary Fig. S8). At the molecular level, alpelisib effectively suppressed p-ERK and p-AKT^(S473)^ levels in both c-Met/H1047R and c-Met/E545K HCCs. In contrast, alpelisib inhibited p-STAT3^(Tyr705)^ and YAP expression in c-Met/H1047R HCC, but not in c-Met/E545K corresponding lesions (Fig. 2f; Supplementary Figs. S12 and S17a). When treating with 20 μM alpelisib the GOF *PIK3CA*-transfected HCC cells, the drug repressed p-STAT3^(Tyr705)^ and YAP levels in H1047R cells, but not E545K cells (Supplementary Fig. S33). The results indicate that p-STAT3^(Tyr705)^ and YAP inhibition might be involved in alpelisib dependent antitumor activity against HCC. Additional studies are required to address this critical issue.

Another important conclusion of the study is that alpelisib suppressed HCC angiogenesis in all the mouse models tested. Notably, even in c-Met/sgPten HCC lesions, where alpelisib demonstrated no efficacy, the treatment led to a decreased microvascular density. Thus, the results indicate that PI3K signaling is necessary for efficient angiogenesis in HCC. On the other hand, the data suggest that suppressing angiogenesis alone is insufficient to cause tumor regression. This observation is consistent with previous reports [24]. Nevertheless, as increased angiogenesis is essential for tumor progression, one can imagine that alpelisib could be combined with other drugs for HCC treatment, and alpelisib anti-angiogenesis activity might contribute to the antitumor effects of the combination therapy.

At the molecular level, alpelisib treatment effectively inhibited AKT and the downstream mTOR pathway in vitro. However, in mouse HCC models, alpelisib treatment could not suppress the mTOR cascade despite the effective inhibition of p-AKT. These findings imply that additional pathways modulate mTOR activation independent of AKT in vivo. Previous studies demonstrated that amino acid levels and the LKB/AMPK pathway could regulate the mTOR cascade [25, 26]. Additional investigations are necessary to unravel how alpelisib modulates these pathways in HCC and how they contribute to alpelisib efficacy in vivo. The present results also highlight the distinct signaling pathways regulated by alpelisib in vitro and in vivo. The differential responses observed are likely because many small molecules effectively inhibit tumor cell growth in culture, yet they have no or limited efficacy in vivo.

Since monotherapy often causes drug resistance during the treatment period, in preclinical experiments or clinical practice, many seek appropriate combination therapies for cancer [27, 28]. Combination treatment could enhance the therapeutic potential of certain drugs and reduce their toxicity by allowing the administration at a lower dose. In the present investigation, we demonstrated that alpelisib synergizes with mTOR or CDK4/6 inhibitors to induce HCC regression in vivo, supporting the further testing of these combination therapies for the treatment of HCCs carrying GOF *PIK3CA* mutations. We chose these molecules based on the pathway analysis using alpelisib monotherapy: alpelisib cannot inhibit the mTOR pathway and has a limited impact on cell cycle regulators. The combination with MLN0128 induced a further inhibition of the PI3K/AKT/mTOR signaling, as evidenced by the significant downregulation of p-mTOR, P-RPS6, and p-4EBP1 levels. Combination with palbociclib led to a pronounced suppression of cell cycle targets, demonstrated by the significantly decreased expression of p-RB, PCNA, CYCLIN A, and CYCLIN D1 (Fig. 8b). Altogether, these findings provide mechanistic insights for alpelisib/mTOR and alpelisib/CDK4/6 combination therapies. To investigate whether the combination treatment is specific for *PIK3CA* mutant HCC, we tested the alpelisib/MLN0128 and alpelisib/palbociclib combination in SNU449 cells without transfection of *PIK3CA* mutations. We found that both combination therapies led to increased tumor cell growth inhibition, although the effects were relatively mild (Supplementary Fig. 34). This contrasted with the H1047R transfected cells, where the combination treatment induced a more profound growth inhibition (Figs. 3 and 4). The results indicate that the combination therapy might be more effective against *PIK3CA* mutant HCCs.

We also tested the therapeutic efficacy of combined alpelisib/cabozantinib treatment in c-Met/H1047R models. In contrast with in vitro studies, the results demonstrated no increased effectiveness of the combination therapy compared to the monotherapy (Supplementary Fig. S30). Further analysis revealed that cabozantinib repressed p-MET and p-ERK expression, implying the efficacy of cabozantinib in this preclinical model [24]. However, it appears clear that both alpelisib and cabozantinib profoundly inhibited the MAPK pathway in vivo, without further suppressing this cascade when administered simultaneously. On the other hand, the combination therapy did not affect the AKT/mTOR cascade. The results indicate that combination therapy targeting distinct signaling pathways is necessary for synergistic antitumor potency. The data also highlight the importance of preclinical studies of combination therapies using mouse models; in vitro cell line studies are insufficient, sometimes providing incorrect conclusions as frequently observed in clinical practice.

## Materials and methods

### Constructs and reagents

The constructs used in this study, including pT3-EF1α-c-Met, pT3-EF1α-HA-PIK3CAH1047R, pT3-EF1α-HA-PIK3CAE545K, pX330-sgPten, and pCMV/sleeping beauty transposase (SB), have been described previously [11, 22, 29]. All the plasmids were purified using the Endotoxin Free Maxi Prep Kit (Sigma–Aldrich) before injection. Alpelisib, MLN0128, palbociclib, and cabozantinib were purchased from LC Laboratories (Woburn, MA). Alpelisib, palbociclib and cabozantinib were formulated in 0.5% Tween 80 and 0.5% carboxymethylcellulose in purified water to a concentration of 5, 20, and 12.5 mg/mL, respectively. MLN0128 was dissolved in NMP (1-methyl-2-pyrrolidinone; Sigma–Aldrich) to generate a concentration of 20 mg/mL. It was diluted 1:200 into 15% PVP/H_2_O (PVP: polyvinylpyrrolidone K 30, Sigma–Aldrich; diluted in H_2_O at a 15.8:84.2 w/v ratio) before administration to mice. All these drugs were dissolved in DMSO for in vitro studies.

### In vitro studies

The human HLE, SNU449, Huh7, SNU-475, SNU387, MHCC97-H, SNU-182, and HepG2 HCC cell lines were used in this study. HLE, Huh7, MHCC97-H, and HepG2 cells were grown in Dulbecco’s modified Eagle medium (DMEM) supplemented with 10% FBS (Gibco) and penicillin (100 U/mL)/streptomycin (100 μg/mL) (Gibco), while SNU449, SNU-475, SNU387, SNU-182 cells were cultured in Roswell Park Memorial Institute 1640 (RPMI 1640) medium supplemented with 10% FBS (Gibco) and penicillin (100 U/mL)/streptomycin (100 μg/mL) (Gibco). Cell lines were maintained at 37 °C and the 5% CO_2_ atmosphere. For IC_50_ values detection, all HCC cells were seeded in 24-well plates and administrated with increasing concentration of drugs in triplicate for 48 h. Subsequently, tests were performed by crystal violet staining. After incubating in lysis solution for 20 min, the diluted solution was added in 96-well plates to measure OD values at 590 nm by the BioTek ELX808 Absorbance Microplate Reader (ThermoFisher Scientific). All experiments were repeated at least three times.

### Hydrodynamic injection and mouse treatment

Female wild-type FVB/N mice were obtained from Charles River Laboratories (Wilmington, MA). The hydrodynamic injection was performed as described previously in detail [30]. Specifically, 20 μg pT3-EF1α-c-Met plasmids and 20 μg pT3-EF1α-HA-PIK3CAH1047R plasmids together with 1.6 μg pCMV/SB in 2 ml of normal saline (0.9%NaCl) were hydrodynamically injected into the tail vein of 6- to 8-week-old FVB/N mice to generate the c-Met/H1047R mouse model. Twenty micrograms pT3-EF1α-c-Met plasmids and 20 μg pT3-EF1α-HA-PIK3CAE545K plasmids together with 1.6 μg pCMV/SB in 2 ml of normal saline (0.9%NaCl) were injected to induce the c-Met/E545K mouse model. Twenty micrograms pT3-EF1α-c-Met plasmids and 20 μg pX330-sgPten plasmids together with 1.6 μg pCMV/SB in 2 ml of normal saline (0.9%NaCl) were injected to establish the c-Met/sgPten mouse model. Alpelisib (25 mg/kg/day), MLN0128 (0.5 mg/kg/day), palbociclib (100 mg/kg/day), cabozantinib (60 mg/kg/day), alpelisib + MLN0128, alpelisib + palbociclib, alpelisib + cabozantinib, or vehicle were orally administered via gavage. Treatments were performed for 3 consecutive weeks after moderate tumor burden occurred. Mice were housed, fed, and monitored following protocols approved by the Committee for Animal Research at the University of California, San Francisco (San Francisco, CA).

### Immunohistochemistry (IHC) and western blot analysis

Liver specimens were fixed in 4% paraformaldehyde and embedded in paraffin. Sections were trimmed at 5 μm in thickness. For immunohistochemistry (IHC), slides were microwaved in 10 mmol/L citrate buffer (pH 6.0) for 10 min for antigen unmasking, followed by a 30 min cool down at room temperature. Subsequently, the slides were incubated in 10% goat serum for 30 min and in Avidin/Biotin solution (Vector Laboratories, Burlingame, CA) for 10 min, respectively. Next, primary antibodies (Supplementary Table S1) were diluted in PBS, applied to the slides, and incubated overnight at 4 °C. Consequently, the slides were subjected to 3% hydrogen peroxide for 10 min, followed by the biotin-conjugated secondary antibody for 30 min at room temperature. The immunoreactivity was detected with the Vectastain Elite ABC kit (Vector Laboratories, Burlingame, CA) and Vector NovaRED™ (Vector Laboratories) as the chromogen. Slides were finally counterstained with hematoxylin solution (ThermoFisher Scientific, Pittsburg, PA).

For western blot analysis, proteins were extracted using the M-PER™ Mammalian Protein Extraction Reagent (ThermoFisher Scientific) together with the Halt™ Protease Inhibitor Cocktail (ThermoFisher Scientific), and concentrations were determined with the Pierce™ Microplate BCA Protein Assay Kit (ThermoFisher Scientific). Proteins were then denatured by boiling in Tris-Glycine SDS Sample Buffer (Bio-Rad). Western blot analysis was performed in SDS-PAGE, and then gels were transferred onto nitrocellulose membranes (Bio-Rad) by electroblotting. After incubation in 10% nonfat milk for 1 h at room temperature, membranes were incubated with primary antibodies (Supplementary Table S1) at 4 °C overnight. The following day, membranes were incubated with horseradish peroxidase-secondary antibody (1:5000; Jackson ImmunoResearch Laboratories Inc., West Grove, PA) at room temperature for 1 h. After three washing steps, membranes were wrapped in plastic and exposed to the X-ray film.

### Determination of the combination index

The combination index (CI) was determined according to the Chou–Talalay method [31]. HCC cells were treated with various concentrations of alpelisib and MLN0128 or palbociclib. Subsequently, the CI was calculated, and CI < 1 indicated a synergistic effect of the combination treatment.

### Statistical analysis

Statistical significance was assessed using the GraphPad Prism 6.0 software (GraphPad Software Inc.). Student’s *t*-test and Tukey–Kramer test were carried where appropriate. Probability (P) values < 0.050 were considered statistically significant.

## Supplementary information

Supplementary Figures

Supplementary Table S1

Related Manuscript File
