# Supplementary material for: Alpelisib combination treatment as novel targeted therapy against hepatocellular carcinoma
Source: Cell Death Dis. 2021 Oct 8;12(10):920. doi: 10.1038/s41419-021-04206-5 (PMC8501067; doi:10.1038/s41419-021-04206-5)
Supplement: Supplementary file 1 — Supplementary Figures [file 41419_2021_4206_MOESM1_ESM.pdf]

**a**

| Dataset | No. HCC cases | No. Mutated cases | %. Mutated cases |
|---|---|---|---|
| TCGA | 366 | 12 | 3.3 |
| MSK | 127 | 2 | 1.6 |
| INSERM | 243 | 4 | 1.6 |
| AMC | 230 | 2 | 0.9 |
| COSMIC | 2147 | 77 | 3.6 |
| Total | 3113 | 97 | 3.1 |

**b**

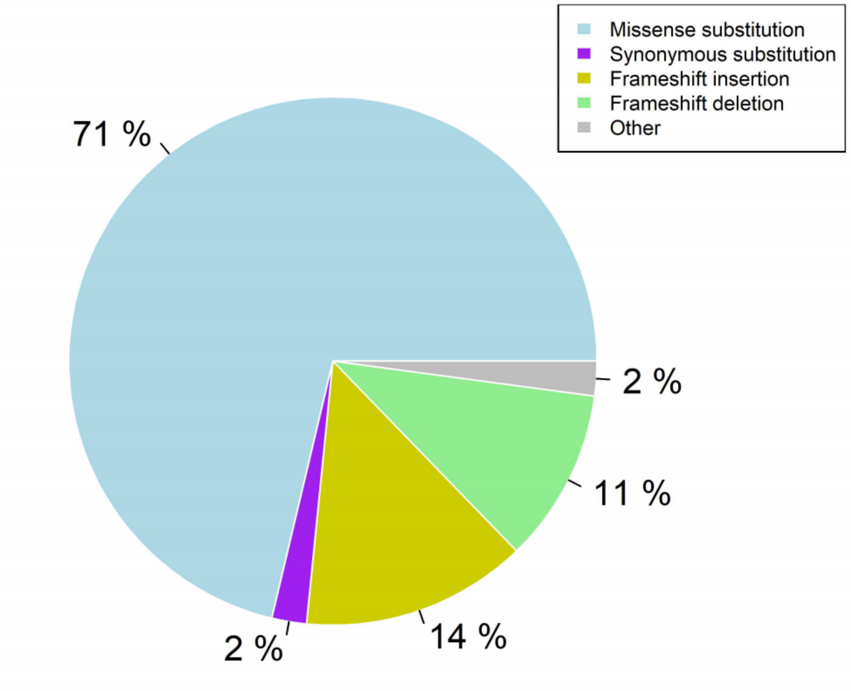


**Supplementary Fig. 1**
*PIK3CA* mutations in human HCC samples. **a,** Analysis of *PIK3CA* mutations in human HCC samples based on TCGA, MSK, INSERM, AMC, and COSMIC databases. **b,** Overview of the *PIK3CA* mutation types of observed in human HCC samples.

a

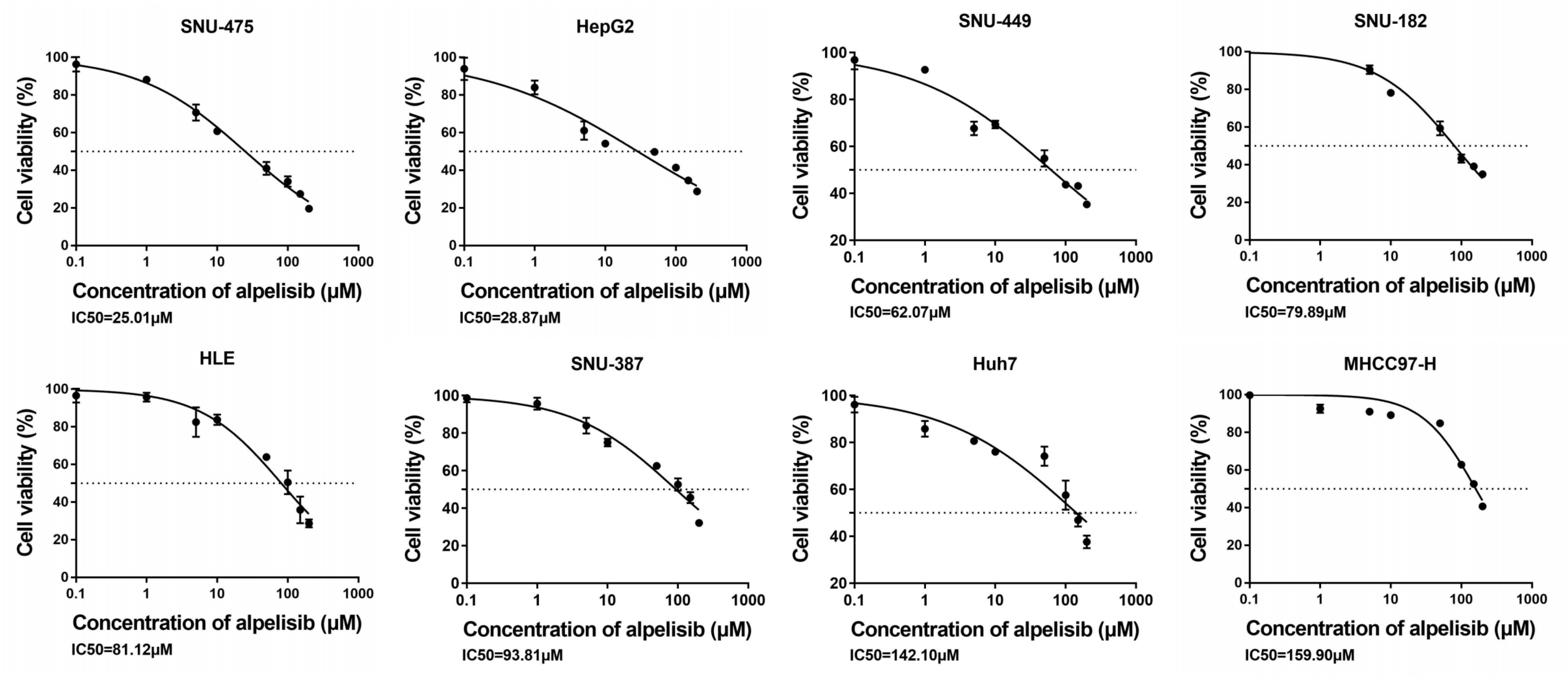


b

| Cell line | SUN-475 | HepG2 | SNU-449 | SNU-182 | HLE | SNU-387 | Huh7 | MHCC97-H |
|---|---|---|---|---|---|---|---|---|
| $IC_{50}$(μM) | 25.01 | 28.87 | 62.07 | 79.89 | 81.12 | 93.81 | 142.10 | 159.90 |

**Supplementary Fig. 2**

Alpelisib inhibits the proliferation of HCC cell lines. **a,** Eight cell lines were treated with escalating concentrations of alpelisib for 48 hours, and $IC_{50}$ values were calculated. **b,** The $IC_{50}$ values of 8 HCC cell lines are listed in the table.

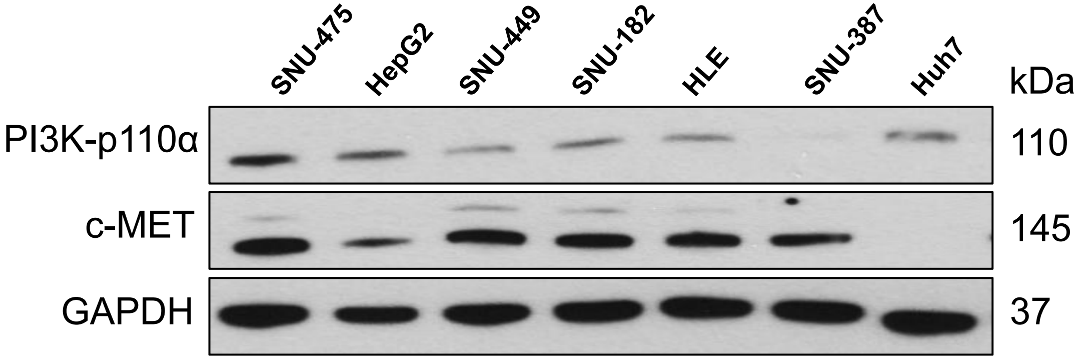


**Supplementary Fig. 3**
Baseline expression status of PI3K and c-MET in HCC cell lines.

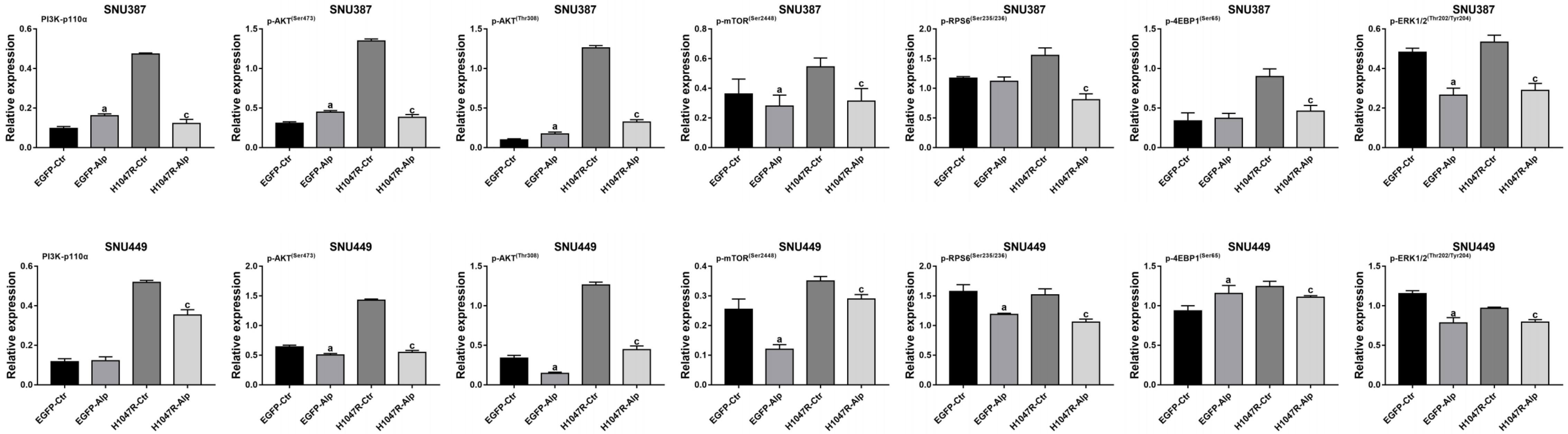


**Supplementary Fig. 4**

Effect of alpelisib on the levels of putative target proteins in SNU387 and SNU449 cell lines. The results of Western blot analysis in Figure 1 were quantified to analyze AKT/mTOR and Ras/MAPK pathways in SNU387 and SNU449 cell lines after treatment with the doses around $IC_{50}$ values of alpelisib for 48 hours. Tukey–Kramer test: at least $P < 0.05$. a, vs EGFP-Ctr; c, vs H1047R-Ctr. Abbreviations: Alp, Alpelisib.

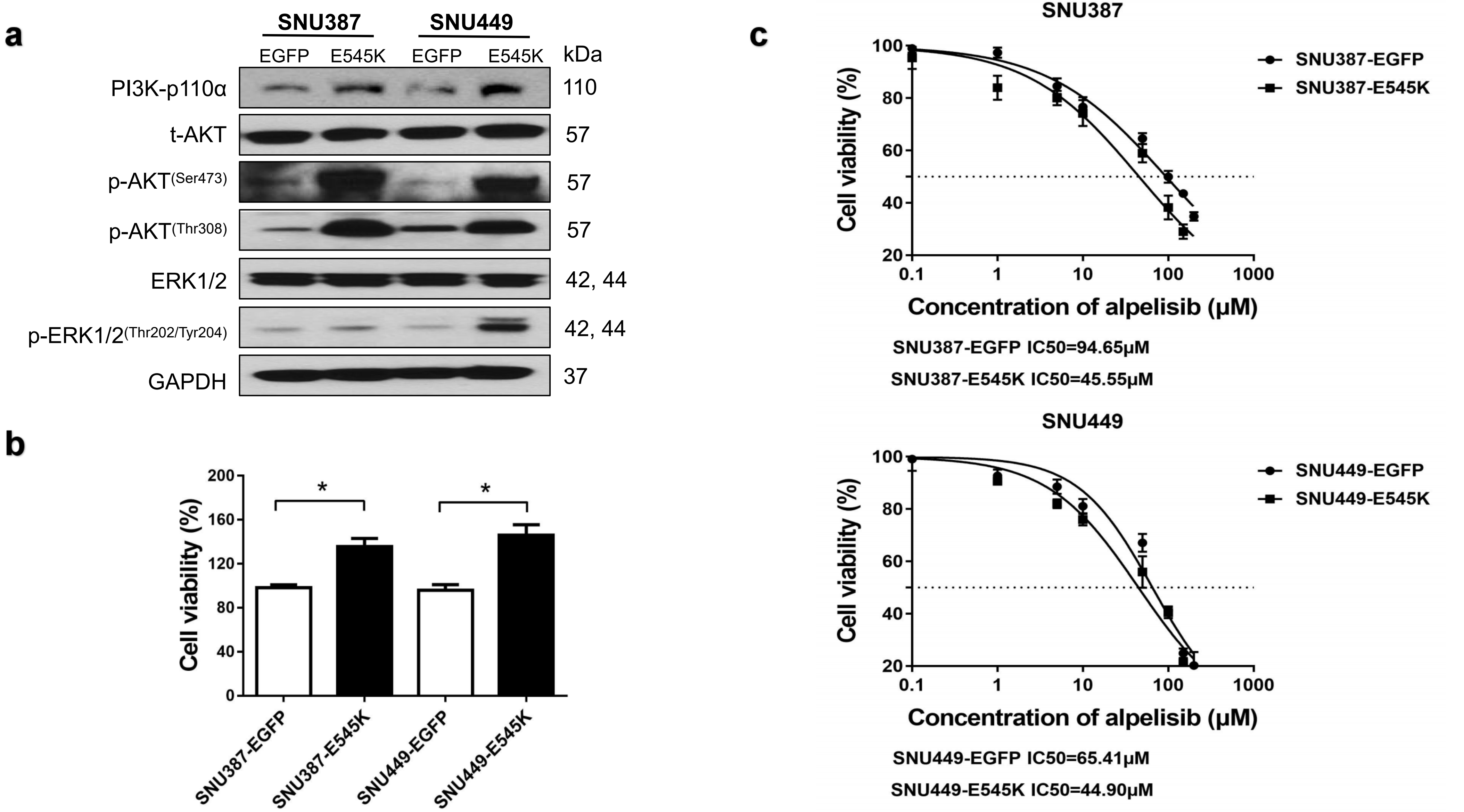


**Supplementary Fig. 5**

Transfection with the *PIK3CAE545K* mutation construct increases the sensitivity to alpelisib treatment of HCC cells. **a,** Transfection with *PIK3CAE545K* mutation construct by lentivirus ad activation of downstream targets in SNU387 and SNU449 cell lines was confirmed by Western blot analysis. **b,** Transfected cell lines were seeded in 24-well plates at $2.5\times10^5$ for 48 hours and cell viability was calculated. **c,** Transfected cell lines were treated with escalating concentrations of alpelisib for 48 hours and $IC_{50}$ values were calculated.

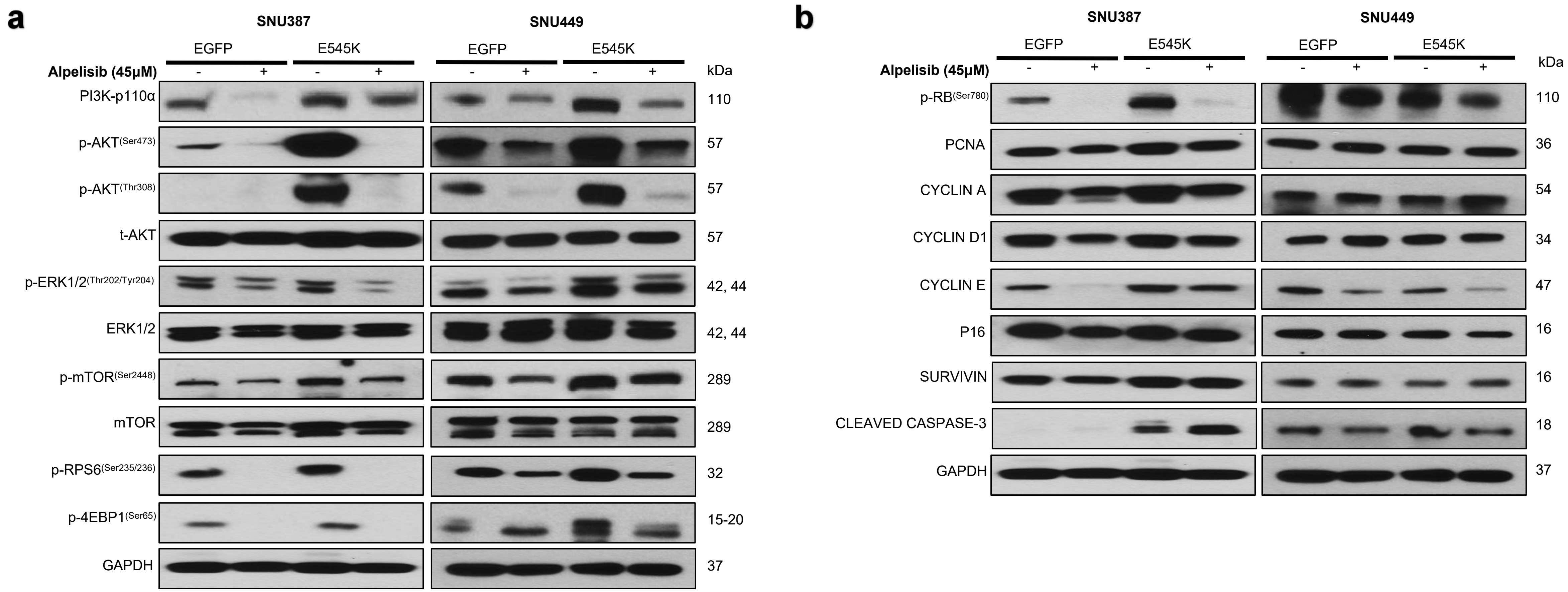


**Supplementary Fig. 6**

Effect of alpelisib on the levels of putative target proteins in SNU387 and SNU449 cell lines. **a,** Western blot analysis of AKT/mTOR and Ras/MAPK pathways after treatment with alpelisib. **b,** Western blot analysis of cell cycle–related proteins after treatment with alpelisib.

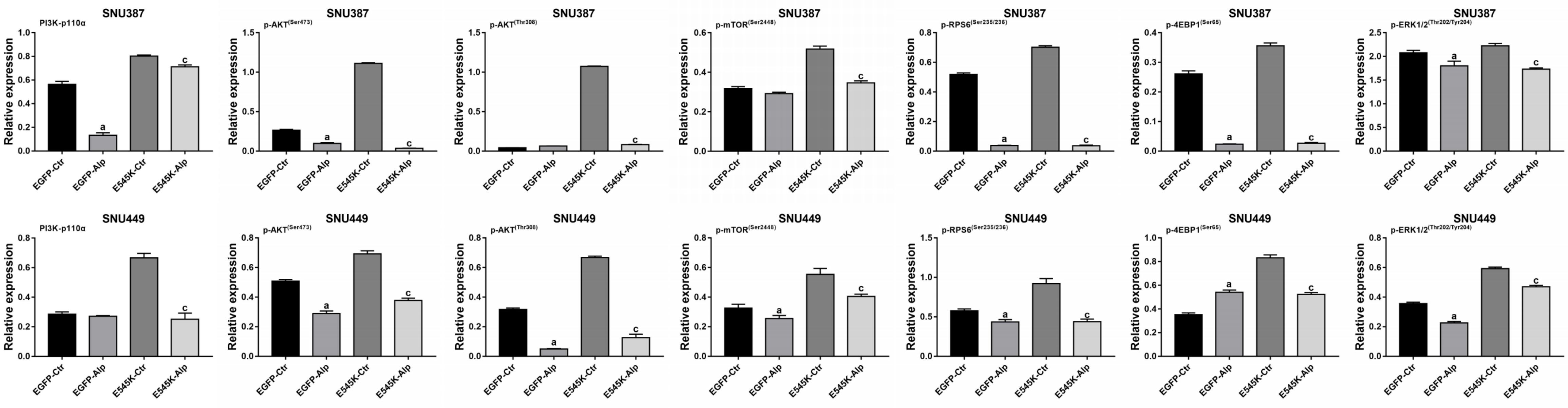


**Supplementary Fig. 7**

Effect of alpelisib on the levels of putative target proteins in SNU387 and SNU449 cell lines. The results of Western blot analysis in Supplementary Figure 5 were quantified to analyze AKT/mTOR and Ras/MAPK pathways in SNU387 and SNU449 cell lines after treatment with the doses around $IC_{50}$ values of alpelisib for 48 hours. Tukey–Kramer test: at least $P < 0.05$. a, vs EGFP-Ctr; c, vs E545K-Ctr. Abbreviations: Alp, Alpelisib.

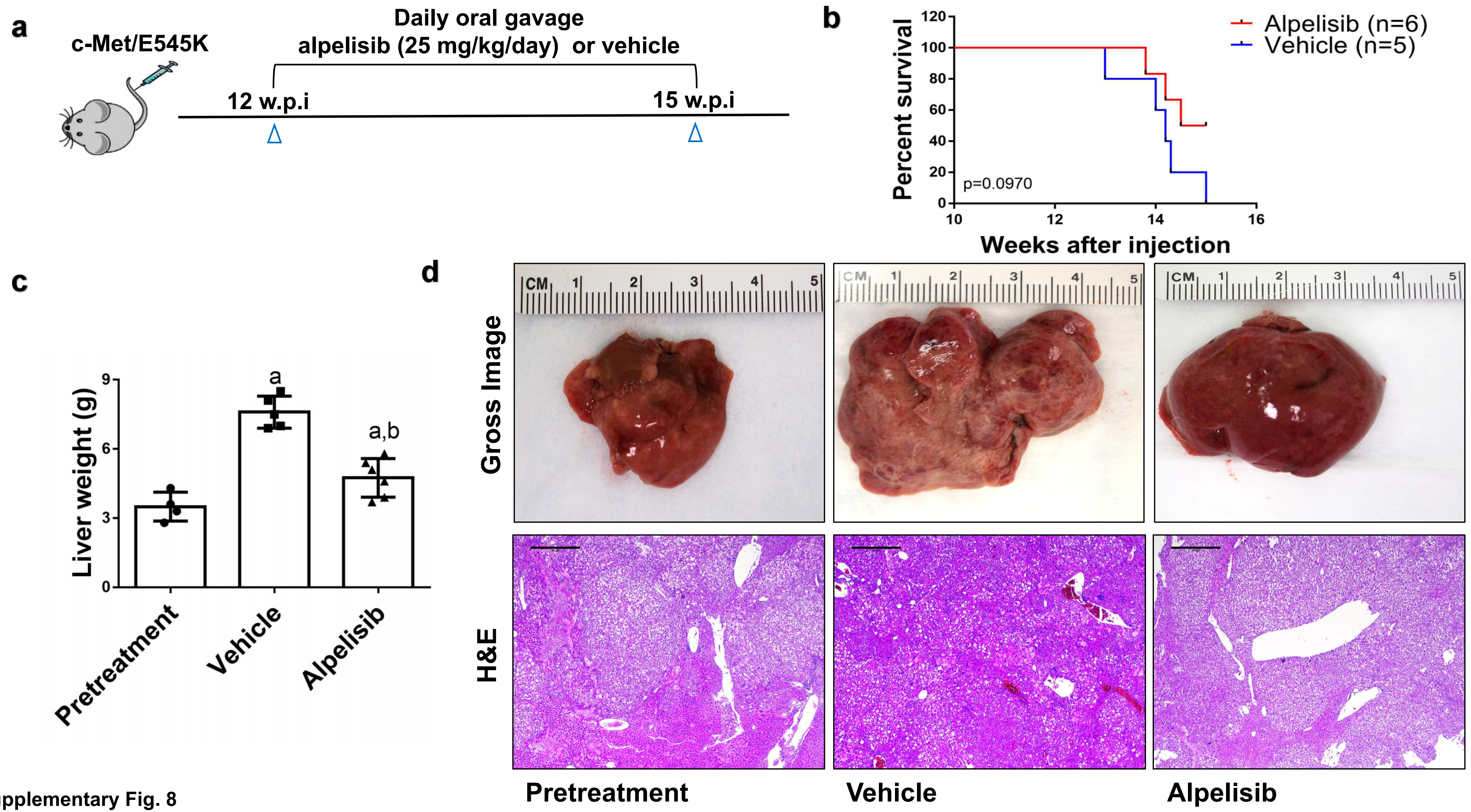


**Supplementary Fig. 8**

Alpelisib treatment has less efficacy in inhibiting the progression of HCC in c-Met/E545K mice. **a,** Study design. w.p.i., weeks post-injection. **b,** Survival curve of c-Met/E545K mice pretreated, treated with alpelisib, and vehicle. **c,** Liver weight of pretreatment, vehicle-, and alpelisib-treated c-Met/E545K mice. **d,** Gross images and H&E staining of livers from pretreatment, vehicle-, and alpelisib-treated c-Met/E545K mice. Magnification ×40; scale bar =500μm.

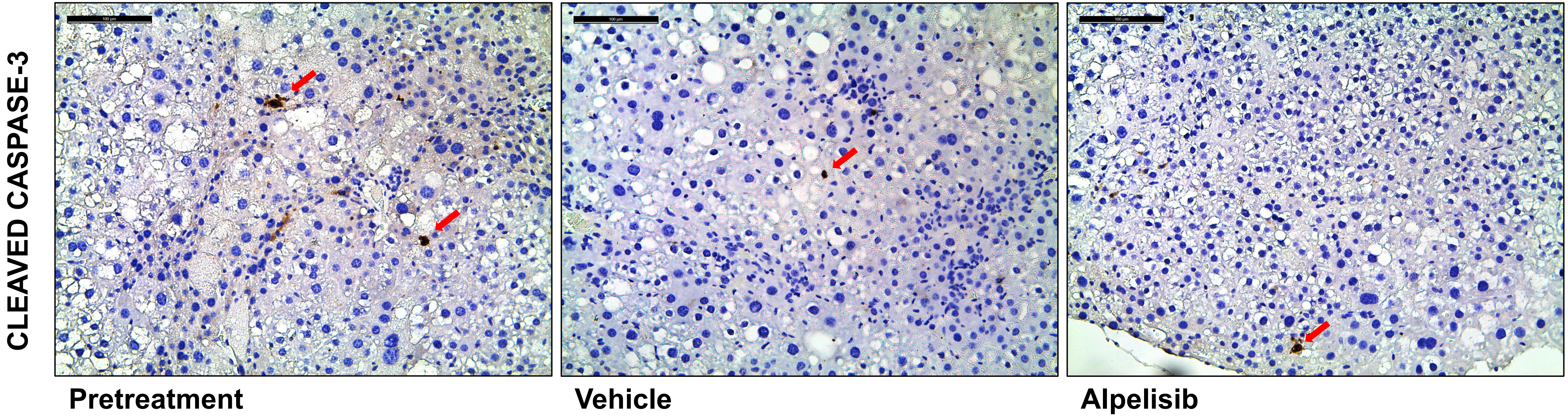


**Supplementary Fig. 9**
CLEAVED CASPASE-3 staining of of livers from pretreatment, vehicle-, and alpelisib-treated c-Met/H1047R mice. Magnification ×200; scale bar =100μm.

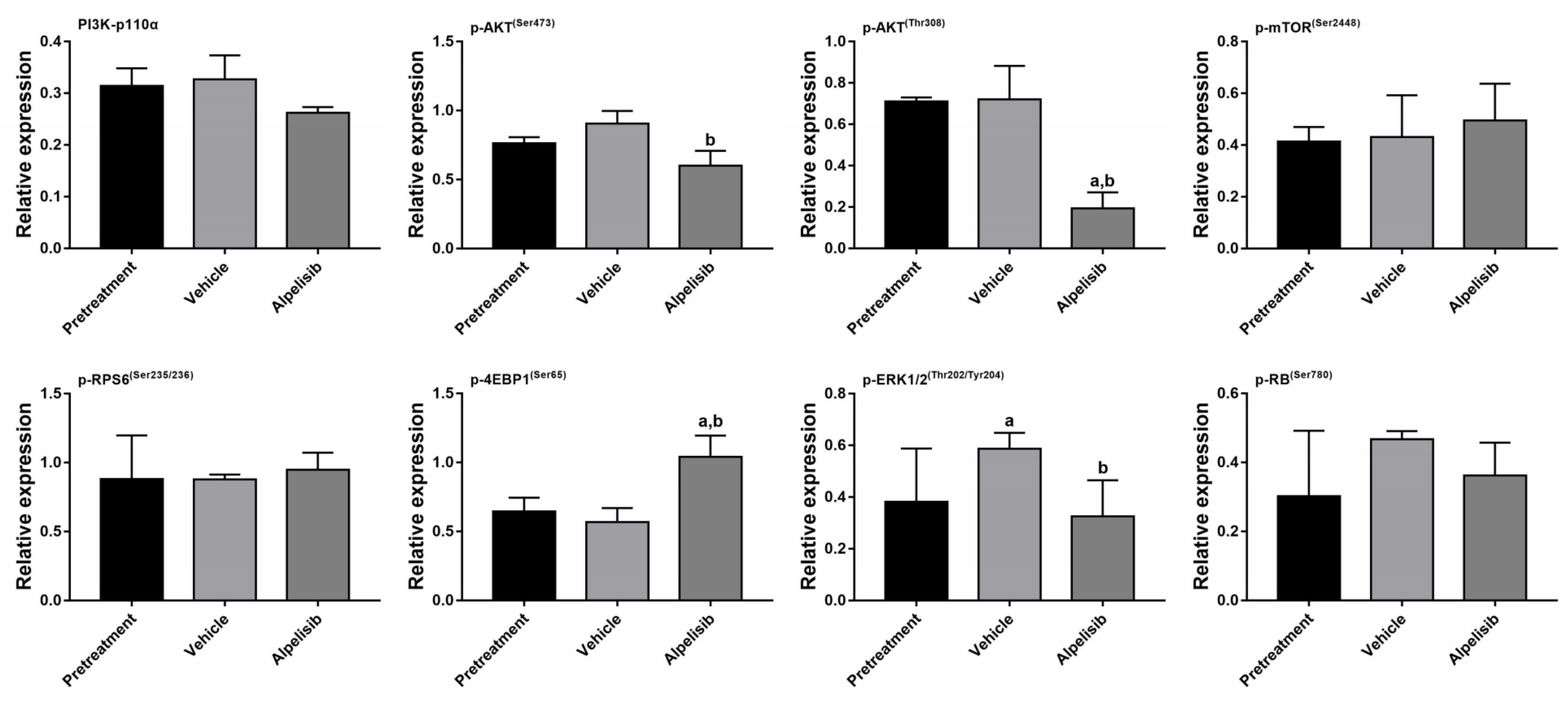


**Supplementary Fig. 10**

Effect of alpelisib on the levels of putative target proteins in c-Met/H1047R mice. The results of Western blot analysis in Figure 2 were quantified to analyze AKT/mTOR and Ras/MAPK pathways in pretreatment, vehicle-, and alpelisib-treated c-Met/H1047R mice. Tukey–Kramer test: at least $P < 0.05$. a, vs. Pretreatment; b, vs. Vehicle.

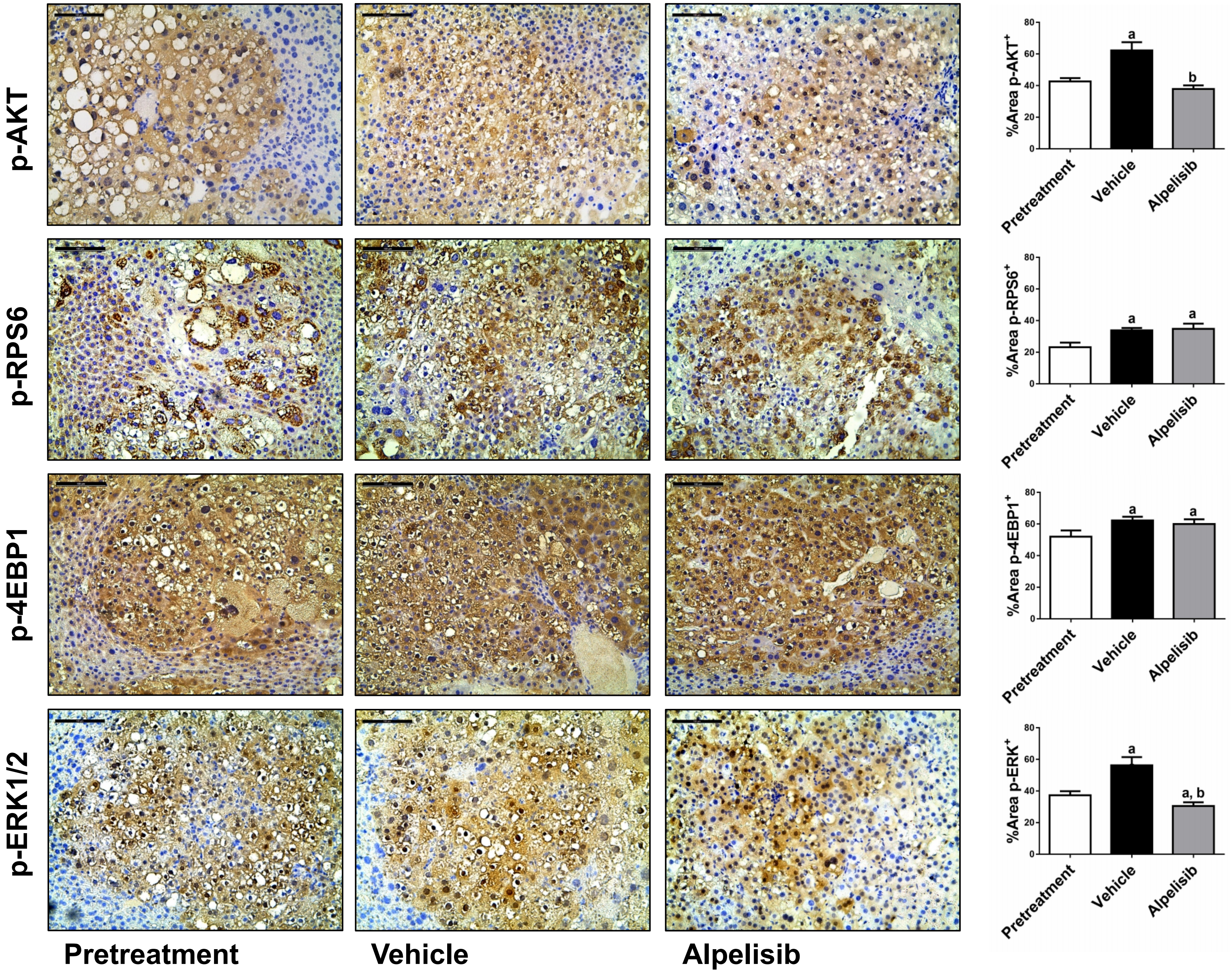


**Supplementary Fig. 11**
Immunohistochemistry of p-AKT, p-RPS6, p-4EBP1, and p-ERK1/2 of livers from pretreatment, vehicle-, and alpelisib-treated c-Met/H1047R mice. Magnification ×200; scale bar =100μm. Tukey–Kramer test: at least $P < 0.05$. a, vs. Pretreatment; b, vs. Vehicle.

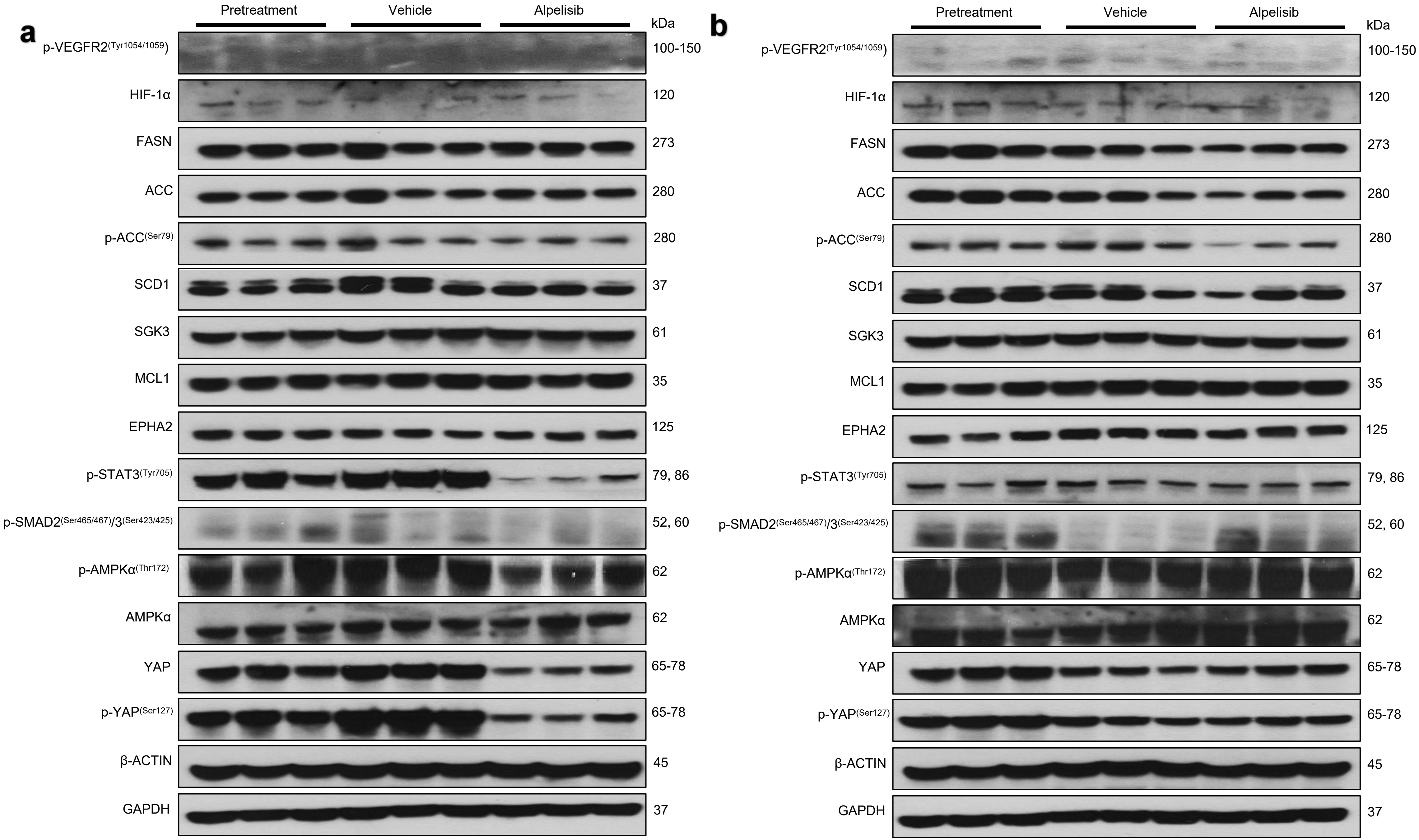


**Supplementary Fig. 12**

Western blot analysis of additional potential targets of alpelisib in pretreatment, vehicle-, and alpelisib-treated c-Met/H1047R mice(**a**) and c-Met/E545K mice (**b**).

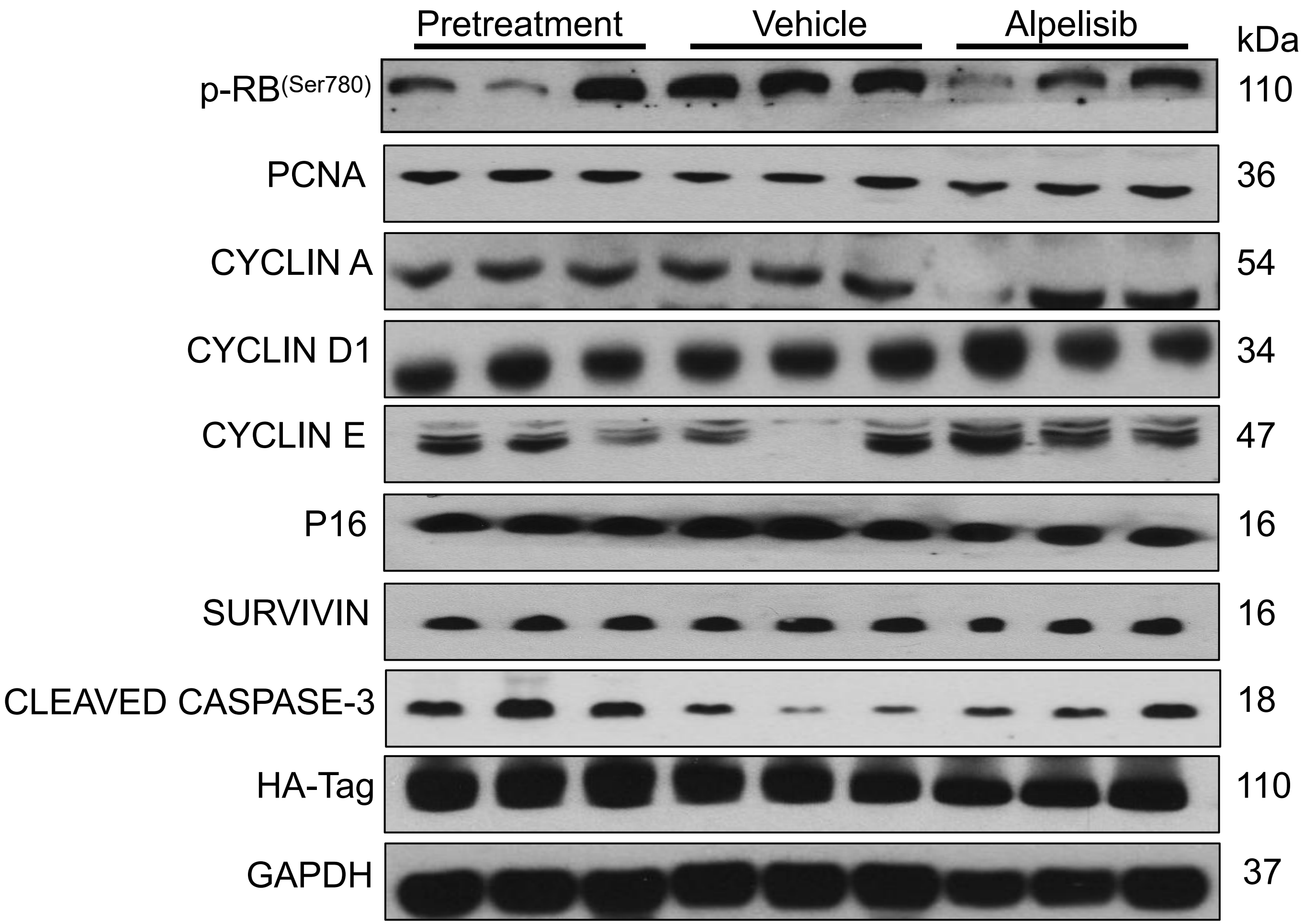


**Supplementary Fig. 13**

Effect of alpelisib on the levels of putative target proteins in c-Met/H1047R mice. Western blot analysis was performed to determine the level of proliferation signaling pathways in pretreatment, vehicle-, and alpelisib-treated c-Met/H1047R mice.

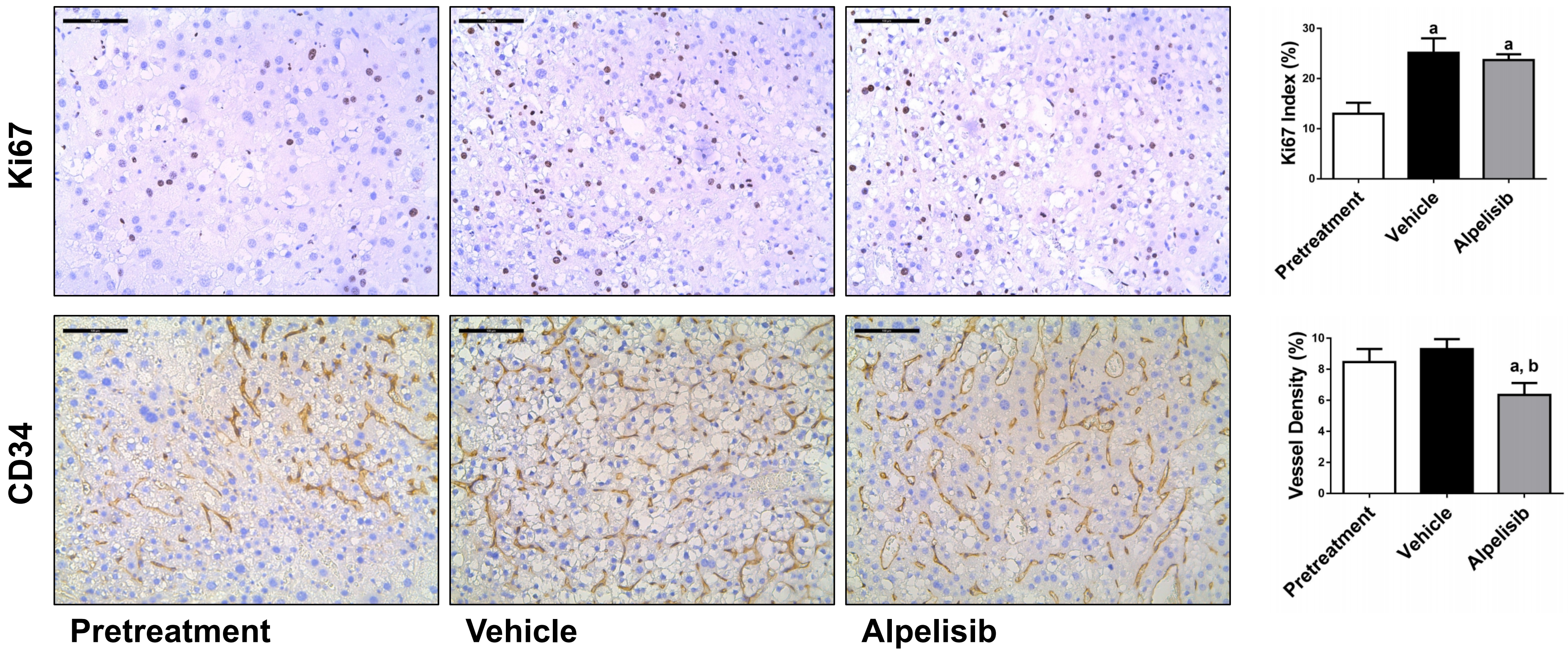


**Supplementary Fig. 14**
Alpelisib treatment has less efficacy in inhibiting the progression of HCC in c-Met/E545K mice. Ki67 (magnification ×200; scale bar =100μm) and CD34 (magnification ×200; scale bar =100μm) staining in livers from c-Met/E545K mice. Ki67-positive cells were counted and quantified as a proliferation index. CD34 staining was quantified and represented as the percentage of the positive staining area of the whole section area. Tukey–Kramer test: at least $P < 0.05$. a, vs. Pretreatment; b, vs. Vehicle.

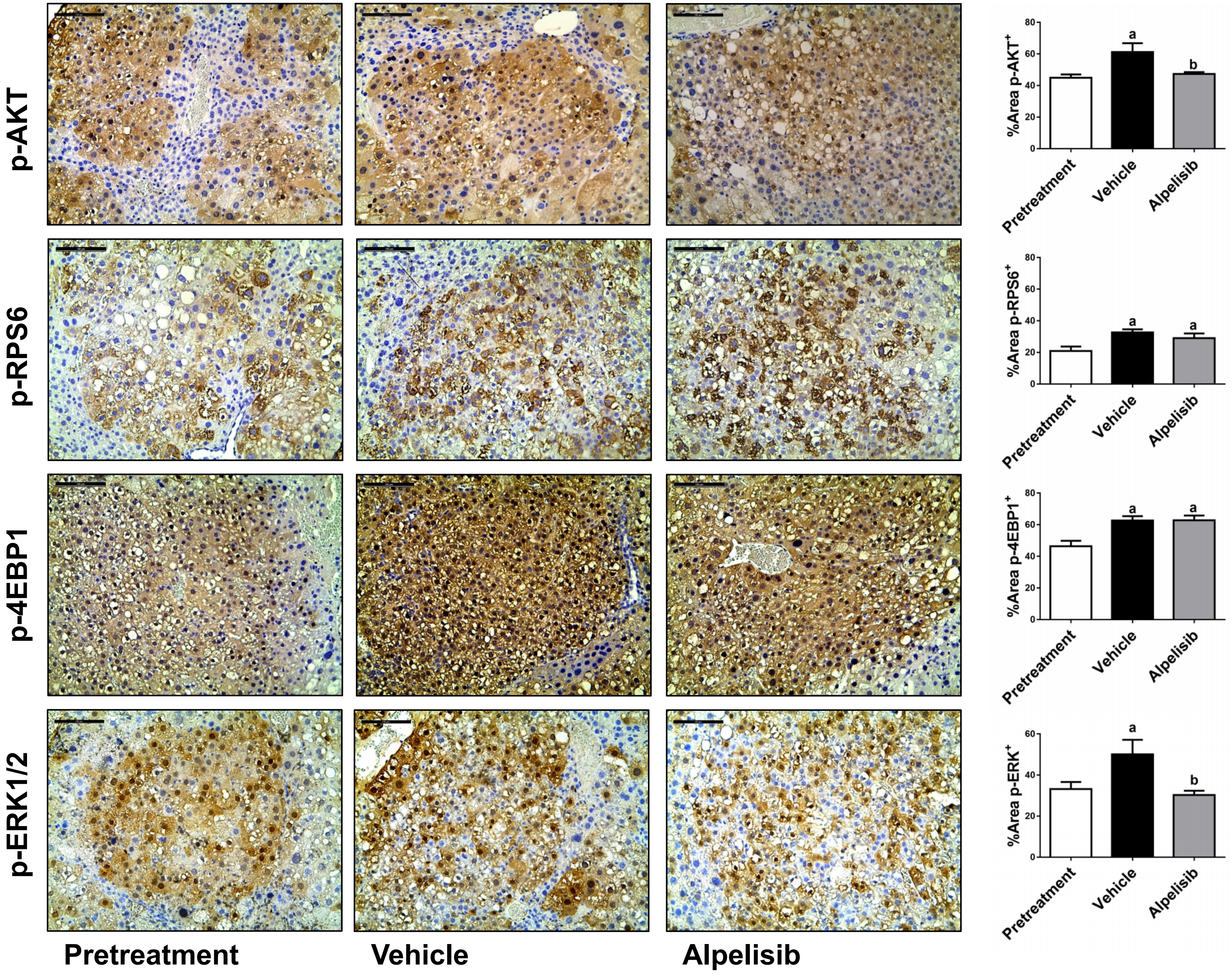


**Supplementary Fig. 15**

Immunohistochemistry staining of p-AKT, p-RPS6, p-4EBP1, and p-ERK1/2 of livers from pretreatment, vehicle-, and alpelisib-treated c-Met/E545K mice. Magnification ×200; scale bar =100μm. Tukey–Kramer test: at least $P < 0.05$. a, vs. Pretreatment; b, vs. Vehicle.

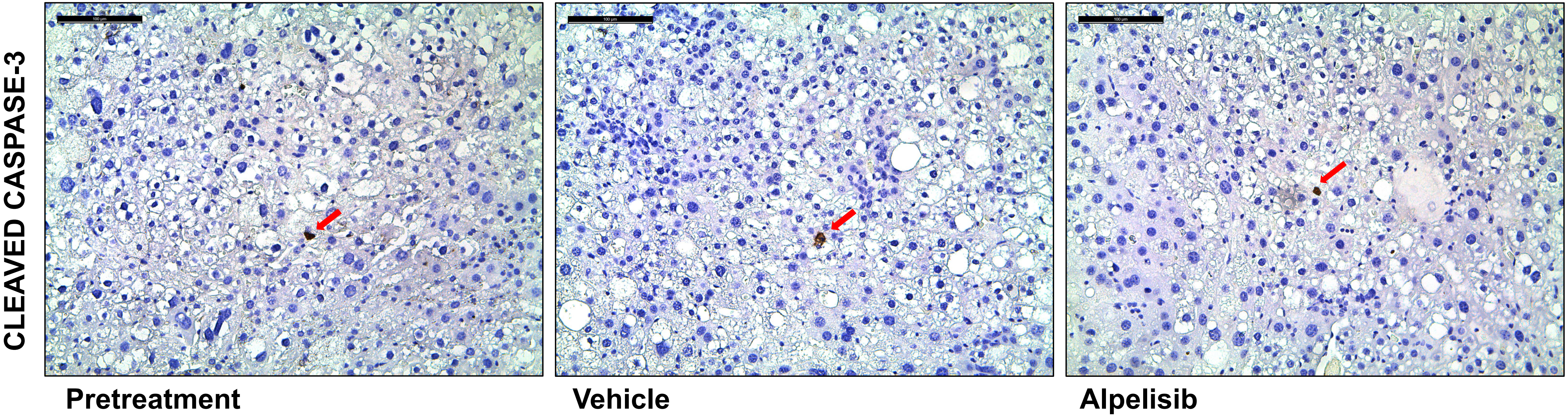


**Supplementary Fig. 16**
CLEAVED CASPASE-3 staining of of livers from pretreatment, vehicle-, and alpelisib-treated c-Met/E545K mice. Magnification ×200; scale bar =100μm.

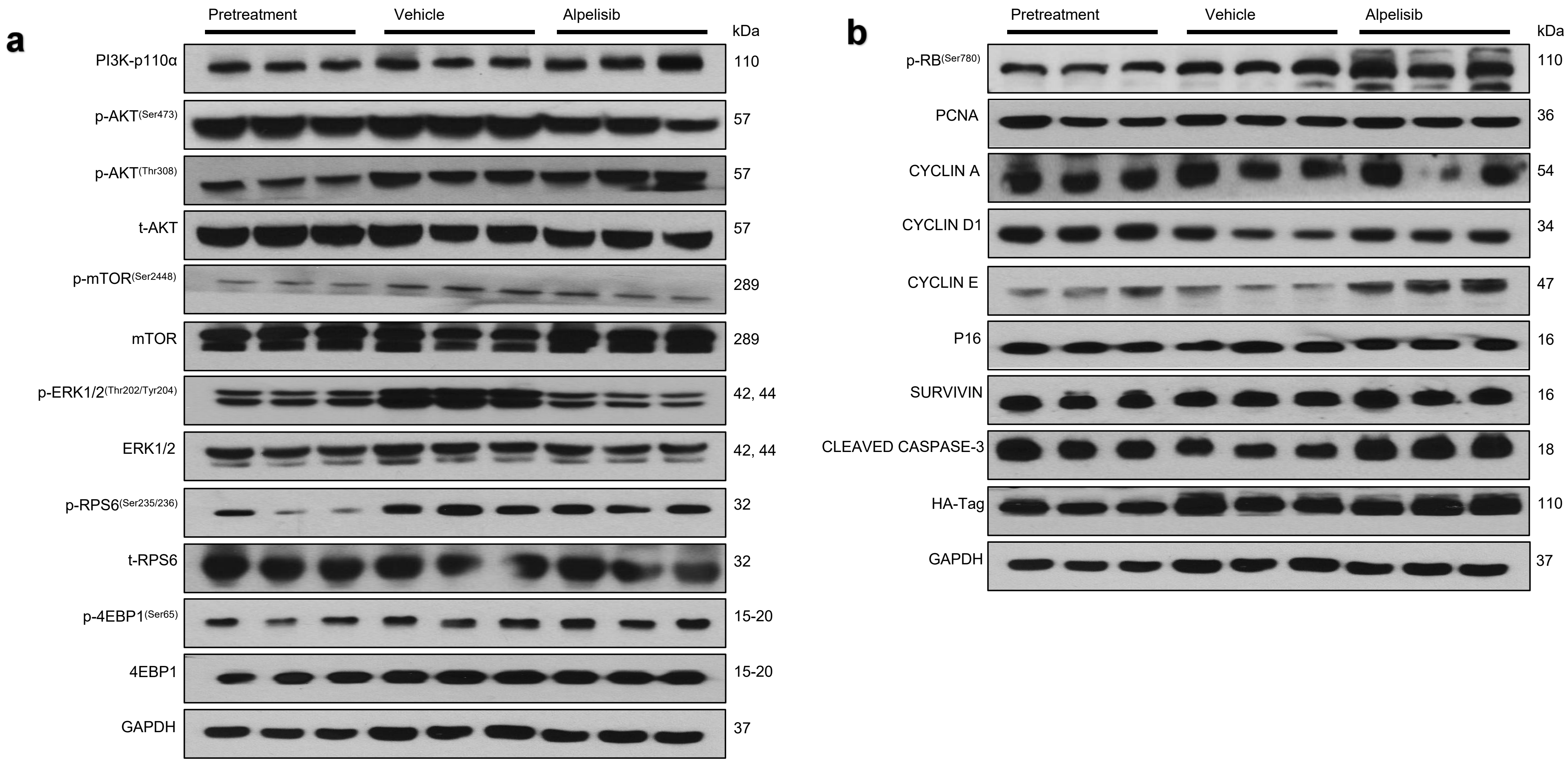


**Supplementary Fig. 17**

Alpelisib treatment has less efficacy in inhibiting the progression of HCC in c-Met/E545K mice. Western blot analysis was performed to determine the level of AKT/mTOR, Ras/MAPK (**a**), and proliferation signaling pathways (**b**) in pretreatment, vehicle-, and alpelisib-treated c-Met/E545K mice.

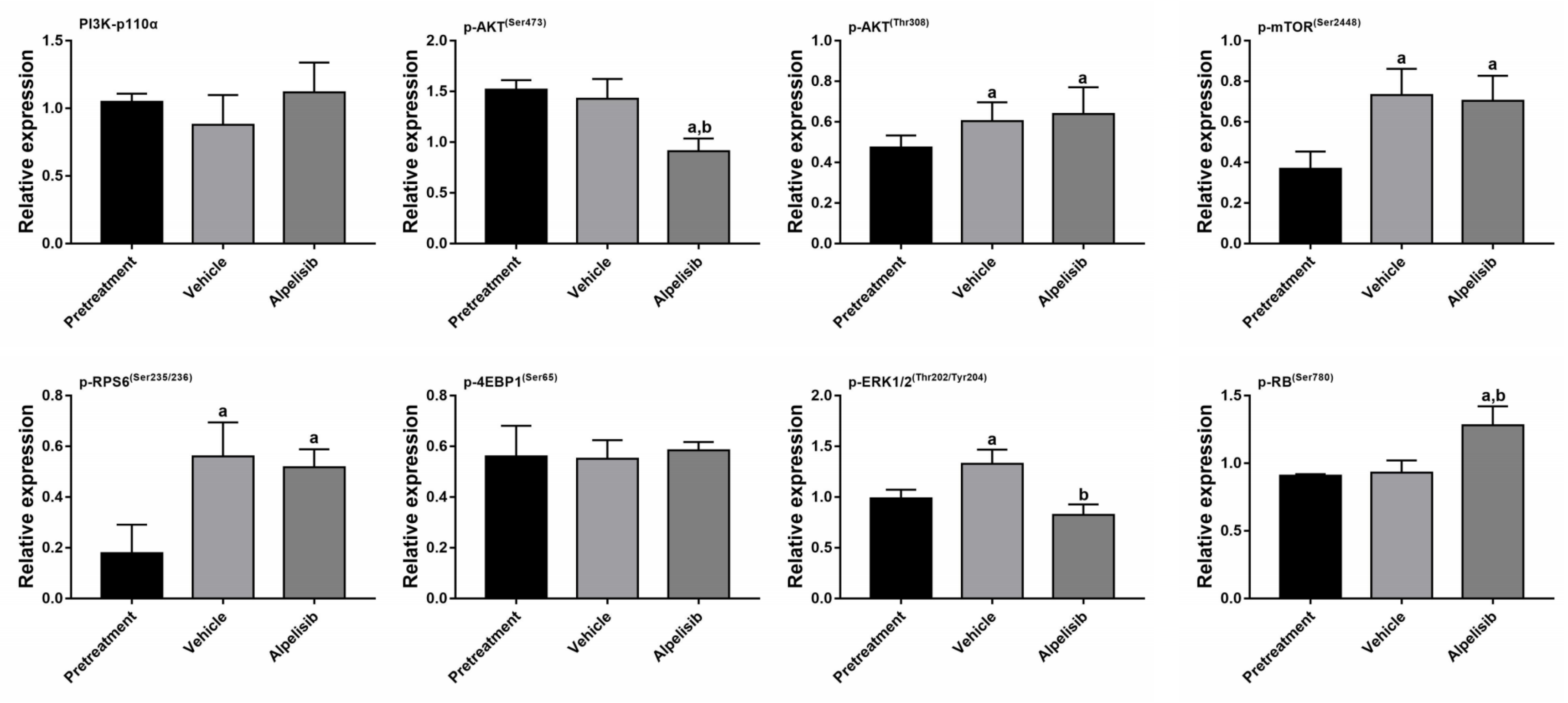


**Supplementary Fig. 18**

Alpelisib treatment has less efficacy in inhibiting the progression of HCC in c-Met/E545K mice. The results of Western blot analysis in Supplementary Figure 16 were quantified to analyze AKT/mTOR, Ras/MAPK and proliferation pathways in pretreatment, vehicle-, and alpelisib-treated c-Met/E545K mice. Tukey–Kramer test: at least $P < 0.05$. a, vs. Pretreatment; b, vs. Vehicle.

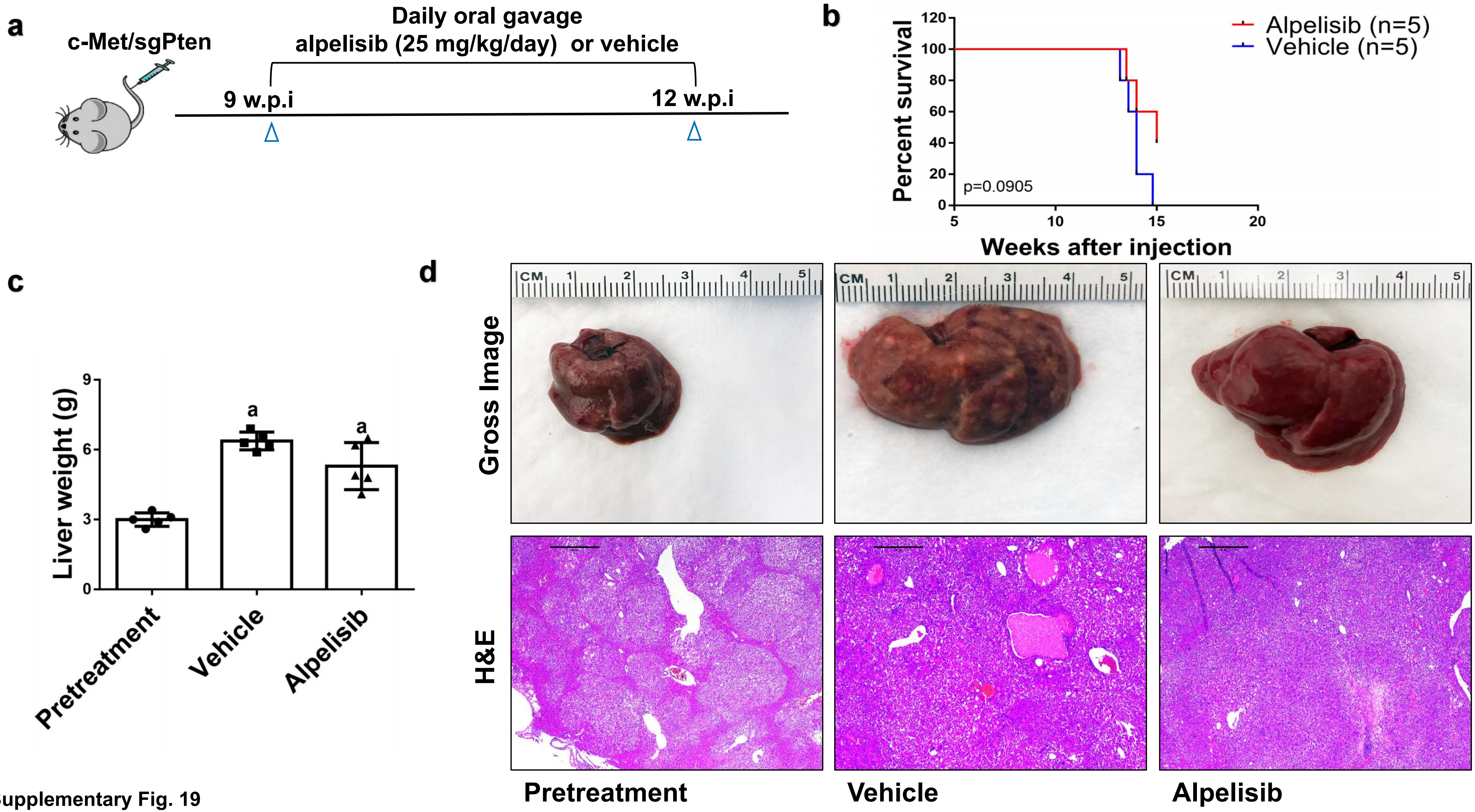


**Supplementary Fig. 19**

Alpelisib treatment has no efficacy in inhibiting the progression of HCC in c-Met/sgPten mice. **a,** Study design. w.p.i., weeks post-injection. **b,** Survival curve of c-Met/sgPten mice pretreated, treated with alpelisib, and vehicle. **c,** Liver weight of pretreatment, vehicle-, and alpelisib-treated c-Met/sgPten mice. **d,** Gross images and H&E staining of livers from pretreatment, vehicle-, and alpelisib-treated c-Met/sgPten mice. Magnification ×40; scale bar =500μm.

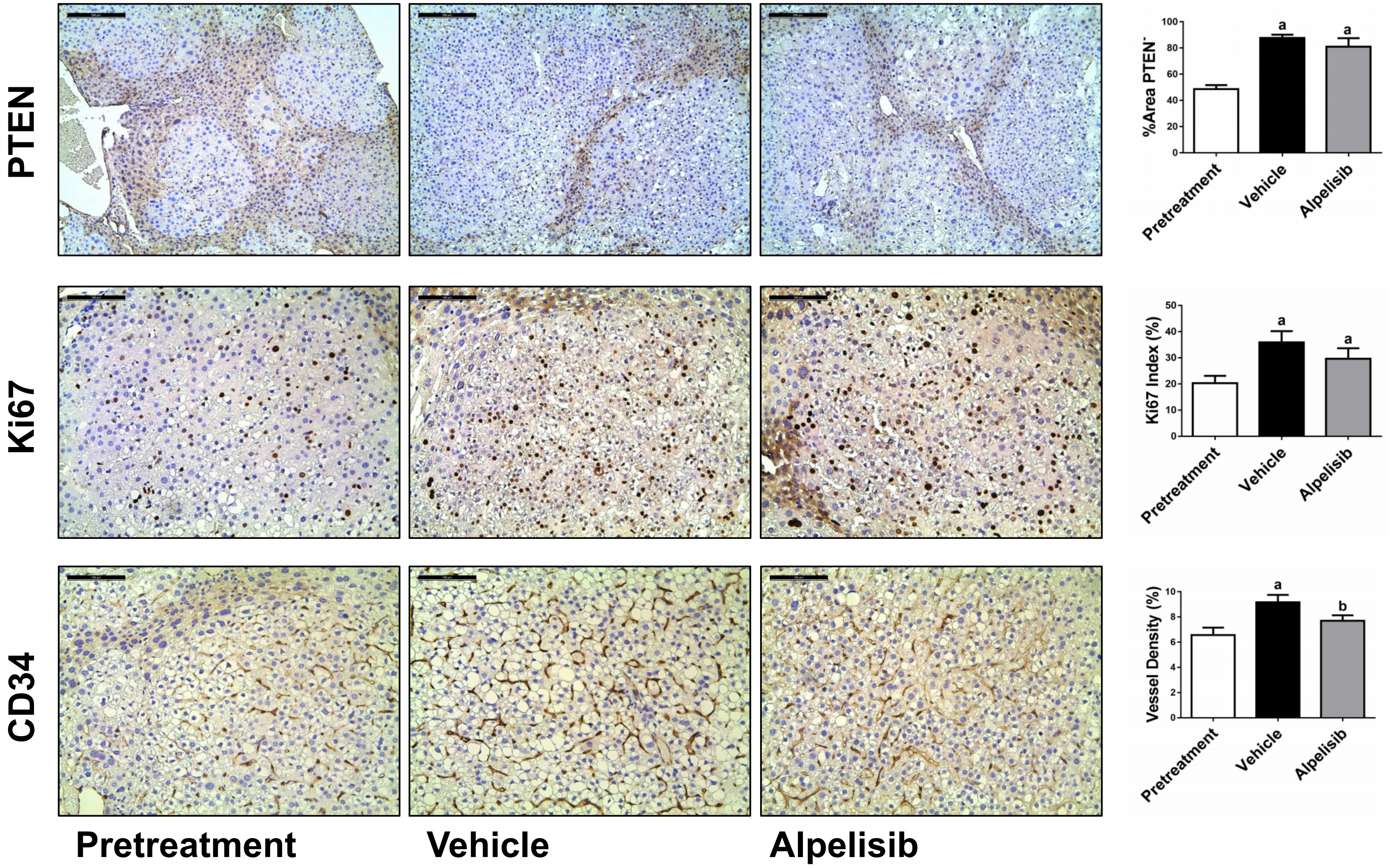


**Supplementary Fig. 20**

Alpelisib treatment has no efficacy in inhibiting the progression of HCC in c-Met/sgPten mice. PTEN (magnification ×200; scale bar =100μm), Ki-67 (magnification ×200; scale bar =100μm) and CD34 (magnification ×200; scale bar =100μm) staining in livers from c-Met/sgPten mice. PTEN staining was quantified and represented as the percentage of the negative staining area of the whole section area. Ki67-positive cells were counted and quantified as proliferation index. CD34 staining was quantified and represented as the percentage of the positive staining area of the whole section area. Tukey–Kramer test: at least $P < 0.05$. a, vs. Pretreatment; b, vs. Vehicle.

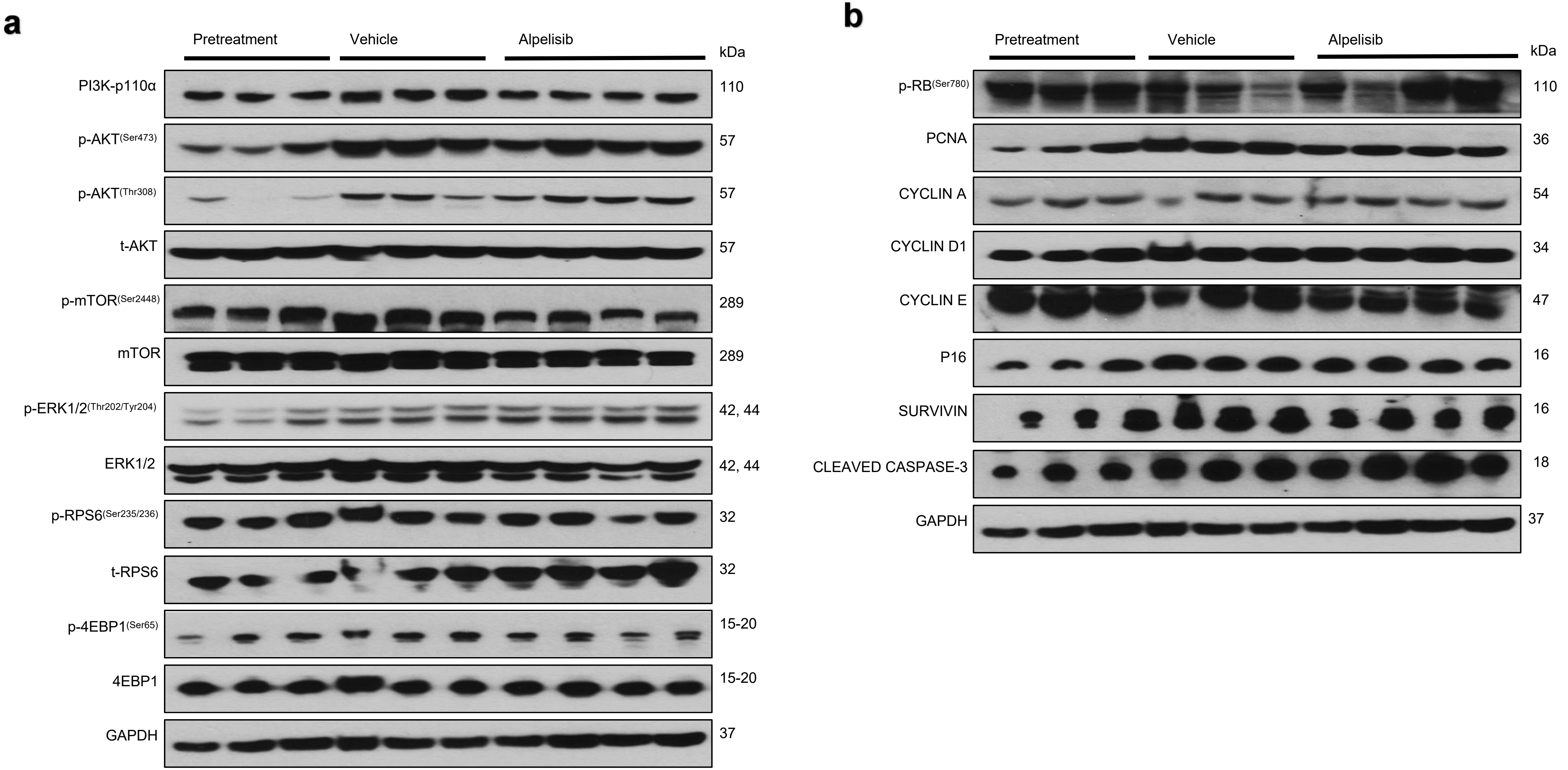


**Supplementary Fig. 21**

Alpelisib treatment has no efficacy in inhibiting the progression of HCC in c-Met/sgPten mice. Western blot analysis was performed to determine the level of AKT/mTOR, Ras/MAPK (**a**), and proliferation signaling pathways (**b**) in pretreatment, vehicle-, and alpelisib-treated c-Met/sgPten mice.

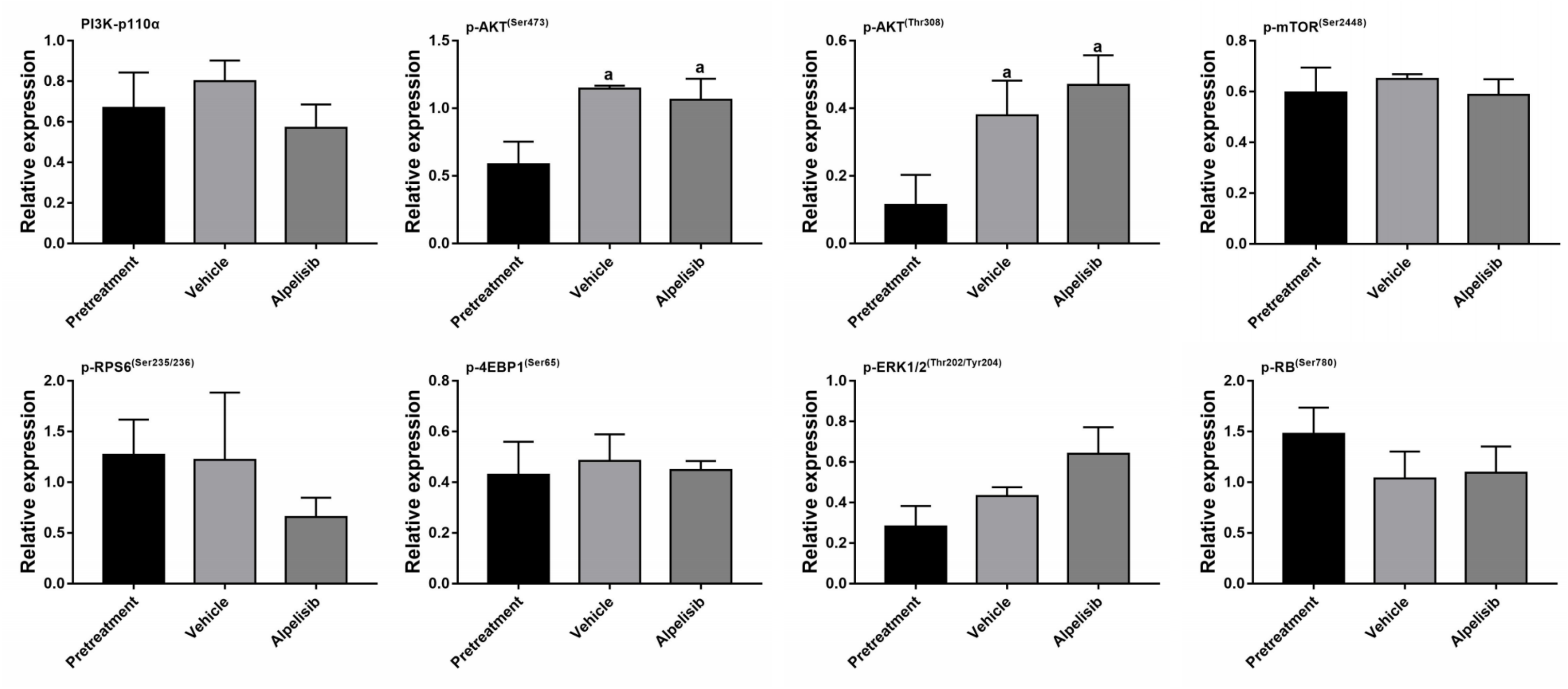


**Supplementary Fig. 22**
Alpelisib treatment has no efficacy in inhibiting the progression of HCC in c-Met/sgPten mice. The results of Western blot analysis in Supplementary Figure 20 were quantified to analyze AKT/mTOR, Ras/MAPK and proliferation pathways in pretreatment, vehicle-, and alpelisib-treated c-Met/sgPten mice. Tukey–Kramer test: at least $P < 0.05$. a, vs. Pretreatment; b, vs. Vehicle.

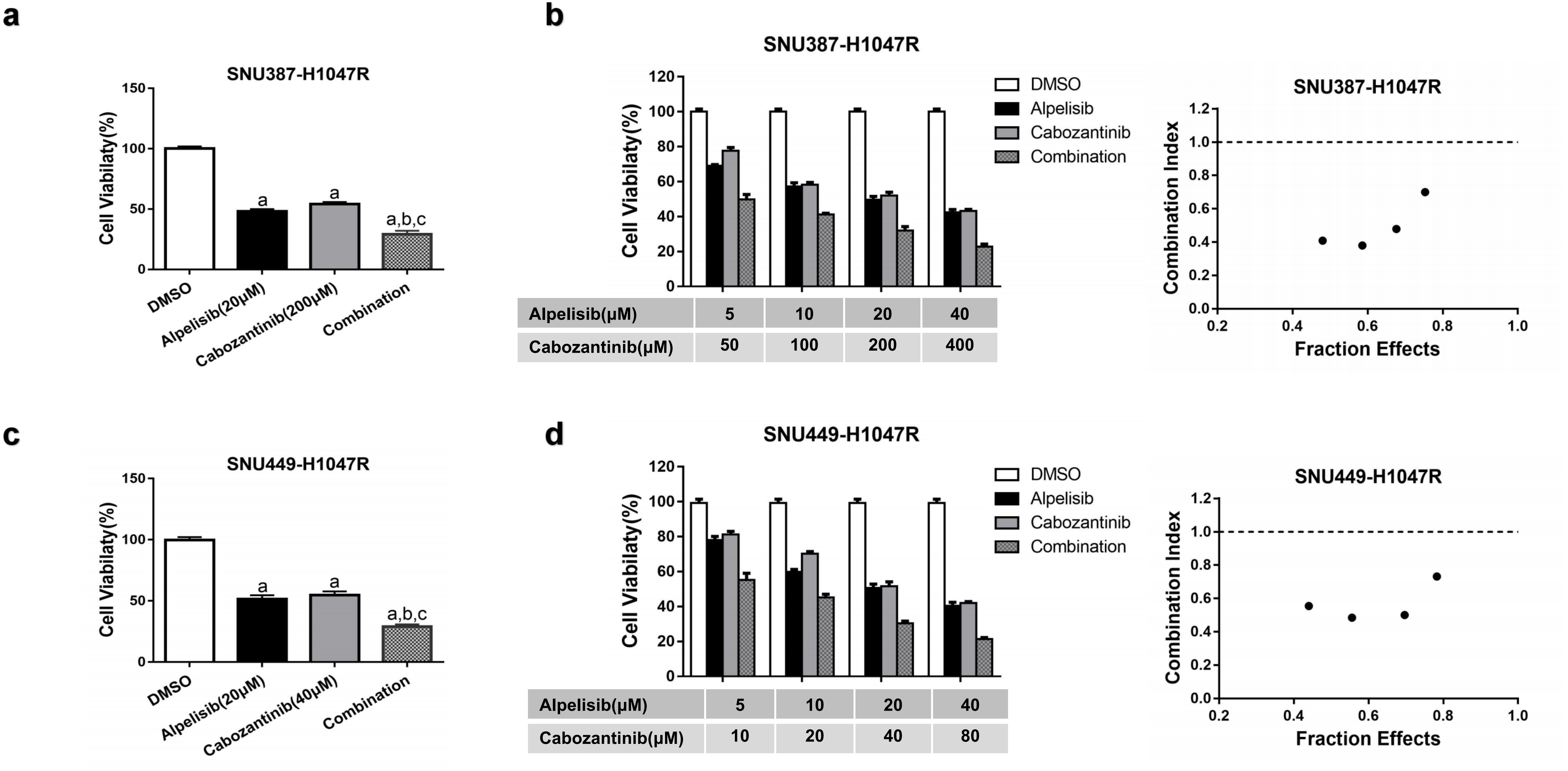


**Supplementary Fig. 23**

Effects of combined alpelisib/cabozantinib treatment on SNU387-H1047R and SNU449-H1047R cell lines. Transfected cell lines were seeded in 24-well plates at $2.5\times10^5$ and treated for 48 hours. **a** and **c,** Combined alpelisib/cabozantinib treatment reduced cell proliferation in SNU387-H1047R and SNU449-H1047R cells. **b** and **d,** The enhanced inhibitory effect of alpelisib/cabozantinib combination on HCC *in vitro* growth is a synergistic action. Tukey–Kramer test: at least $P < 0.05$. a, vs. DMSO; b, vs. Alpelisib; c, vs. Cabozantinib.

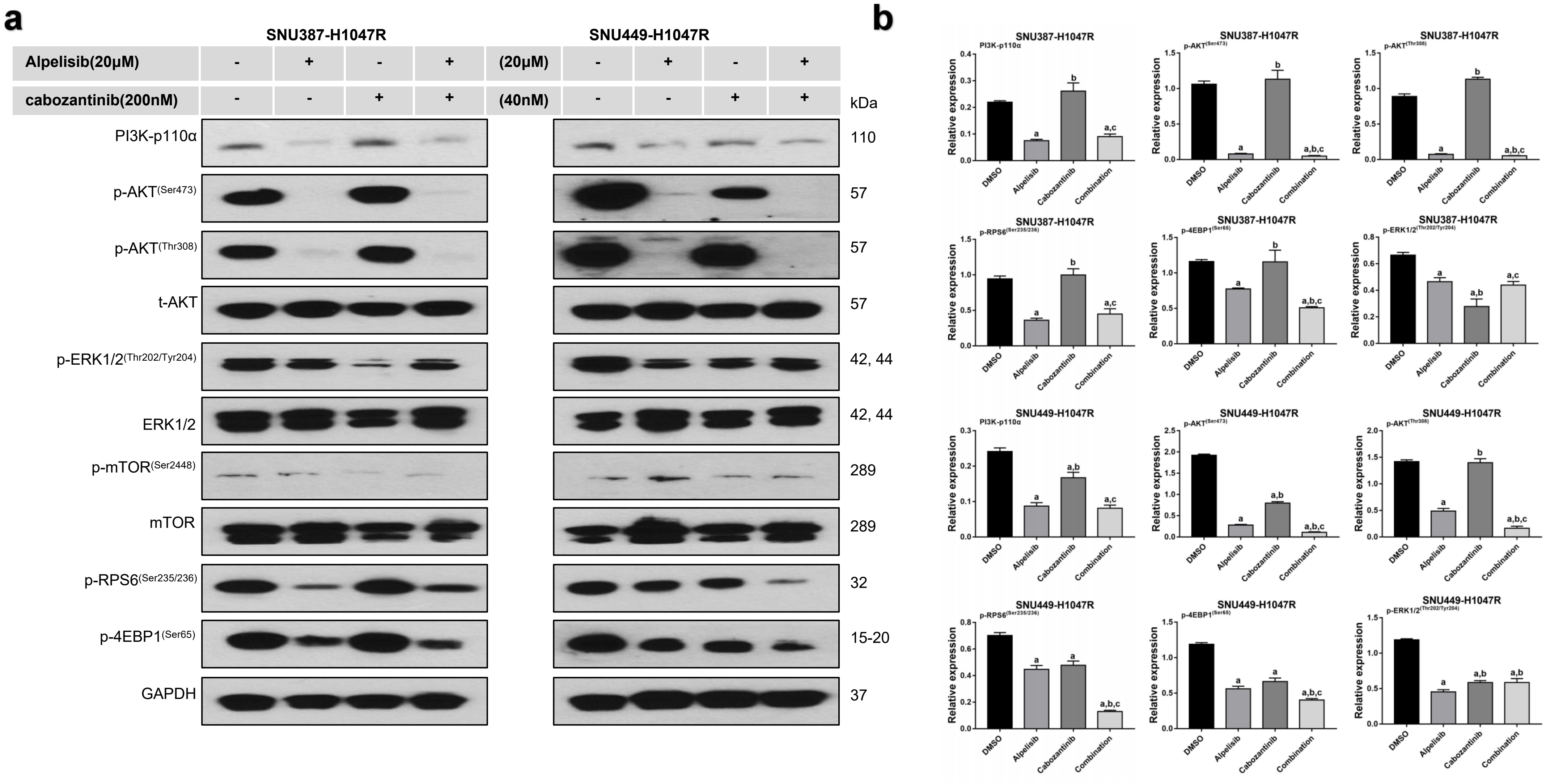


**Supplementary Fig. 24**

Effects of combined alpelisib/cabozantinib treatment on SNU387-H1047R and SNU449-H1047R cell lines. **a,** Western blot analysis of AKT/mTOR and Ras/MAPK pathways in SNU387-H1047R and SNU449-H1047R cell lines. **b,** Quantification analysis of AKT/mTOR and Ras/MAPK pathways in SNU387-H1047R and SNU449-H1047R cell lines. Tukey–Kramer test: at least $P < 0.05$. a, vs. DMSO; b, vs. Alpelisib; c, vs. Cabozantinib.

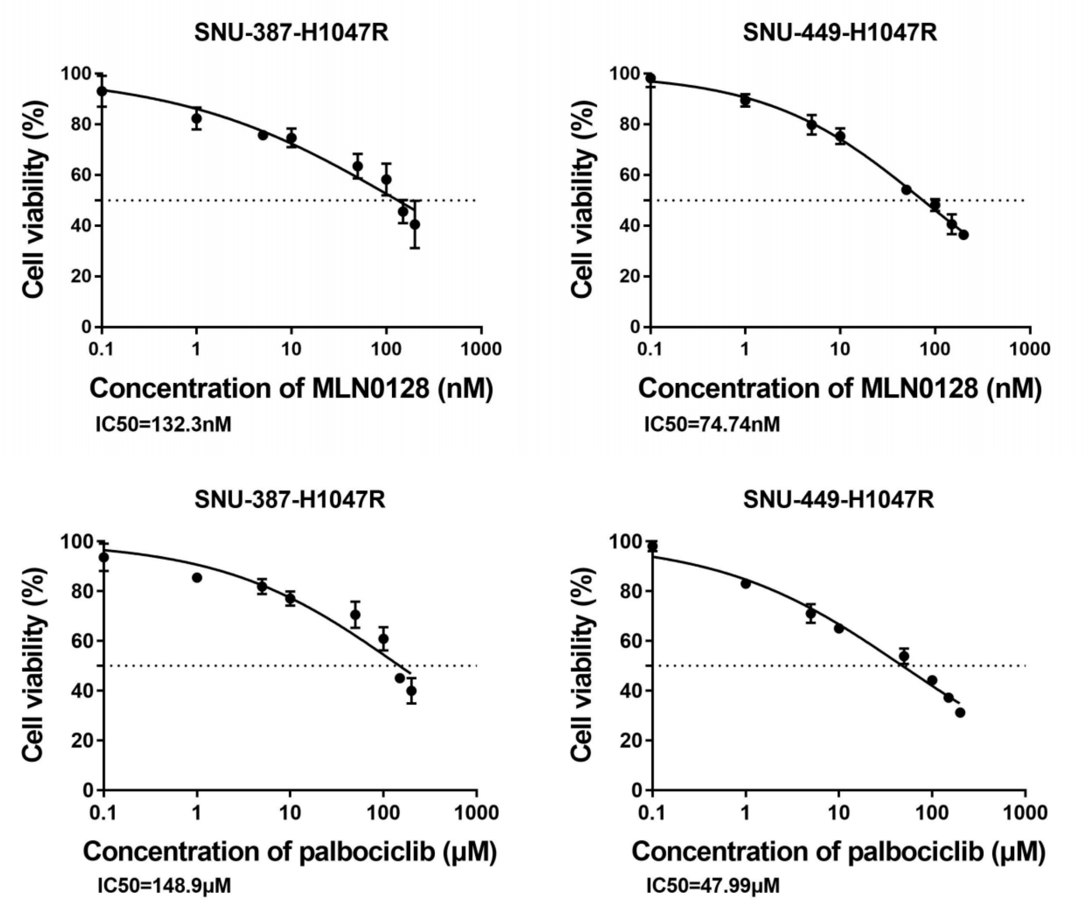


**Supplementary Fig. 25**

Transfected cell lines were treated with escalating concentrations of MLN0128 or palbociclib for 48 hours, and $IC_{50}$ values were calculated.

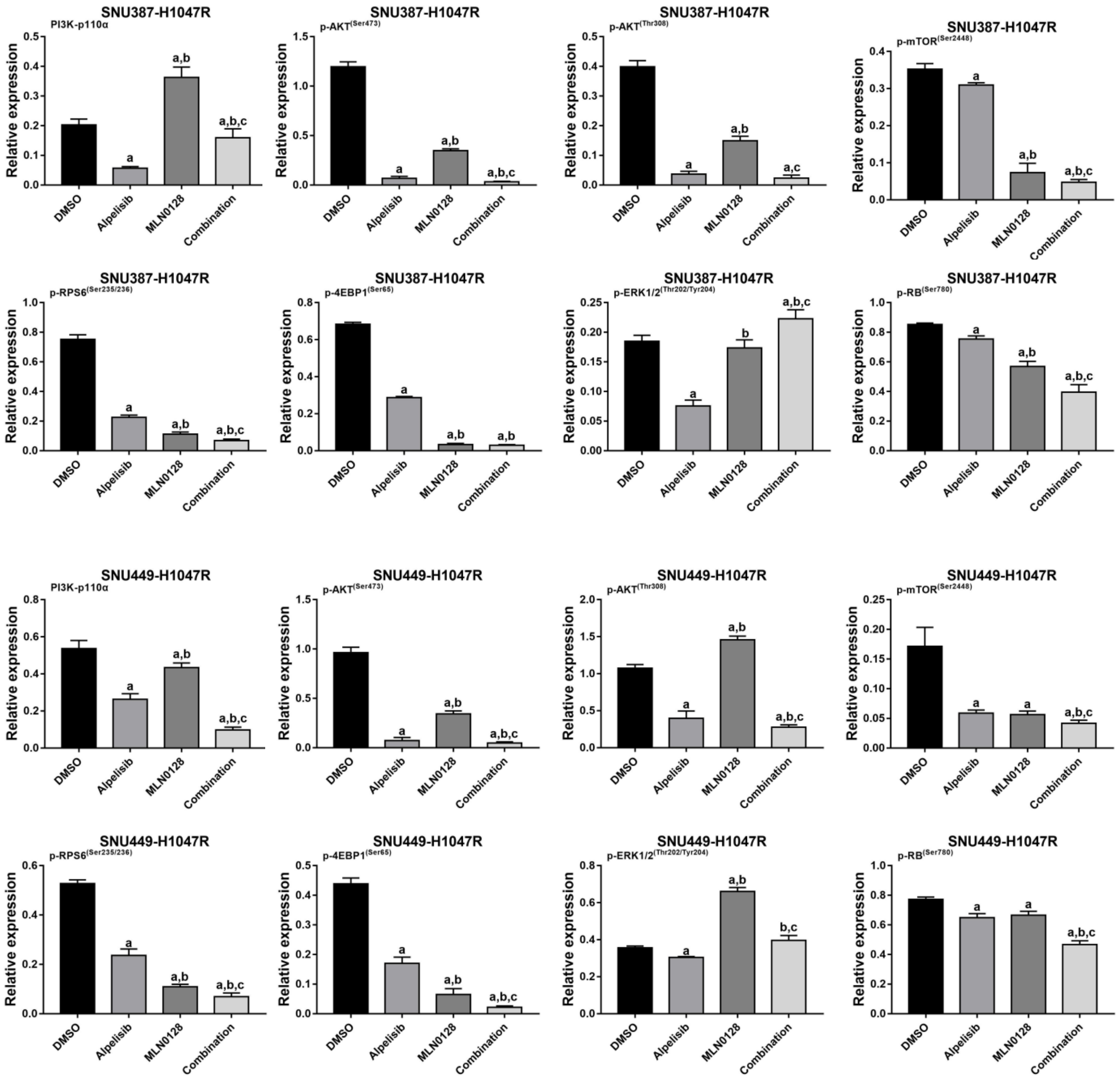


**Supplementary Fig. 26**

Effects of combined alpelisib/MLN0128 treatment on SNU387-H1047R and SNU449-H1047R cell lines. The results of Western blot analysis in Figure 3 were quantified to analyze AKT/mTOR, Ras/MAPK and proliferation pathways in SNU387-H1047R and SNU449-H1047R cell lines. Tukey–Kramer test: at least $P < 0.05$. a, vs. DMSO; b, vs. Alpelisib; c, vs. MLN0128.

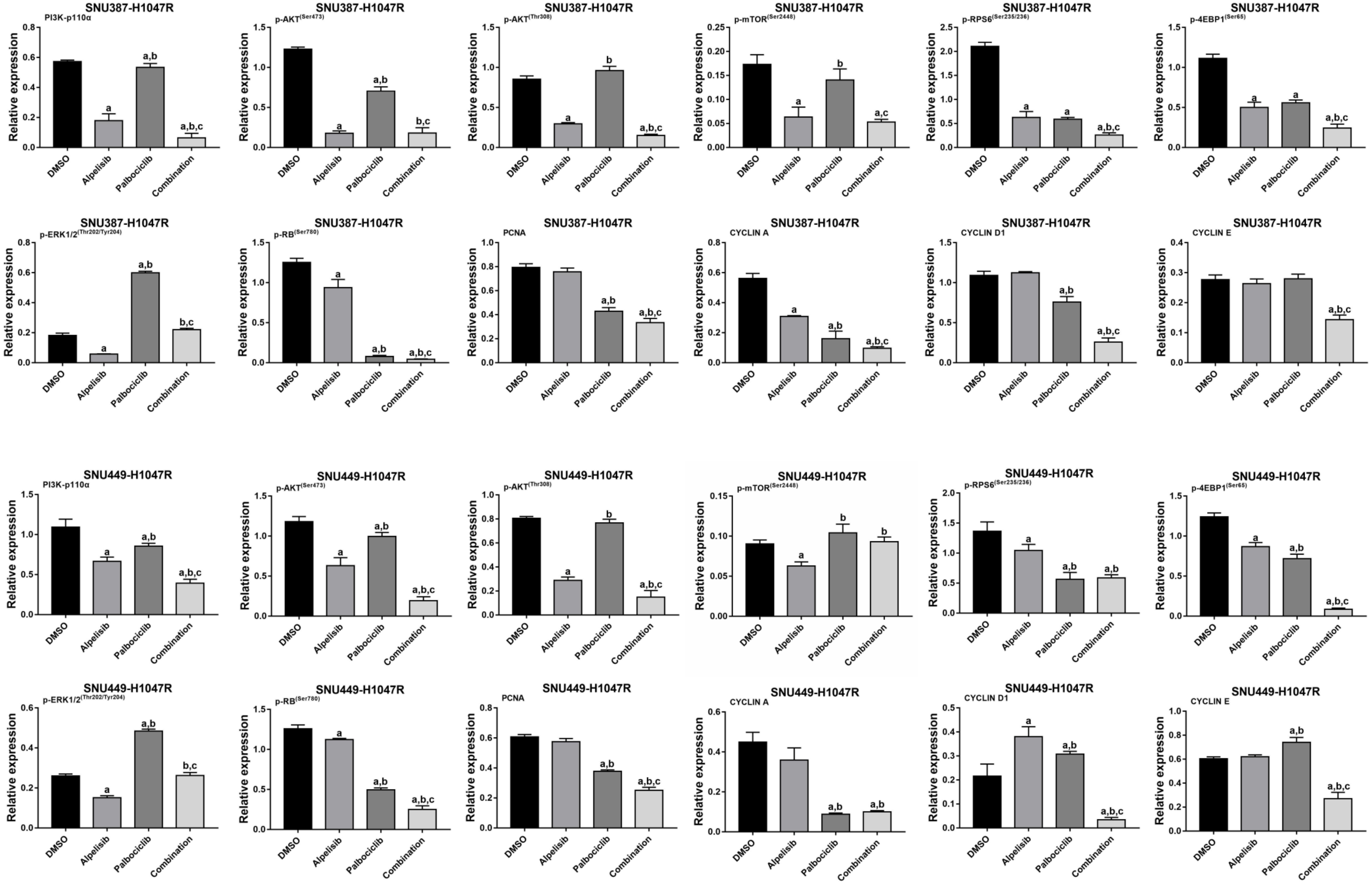


**Supplementary Fig. 27**

Effects of combined alpelisib/palbociclib treatment on SNU387-H1047R and SNU449-H1047R cell lines. The results of Western blot analysis in Figure 4 were quantified to analyze AKT/mTOR, Ras/MAPK and proliferation pathways in SNU387-H1047R and SNU449-H1047R cell lines. Tukey–Kramer test: at least $P < 0.05$. a, vs. DMSO; b, vs. Alpelisib; c, vs. Palbociclib.

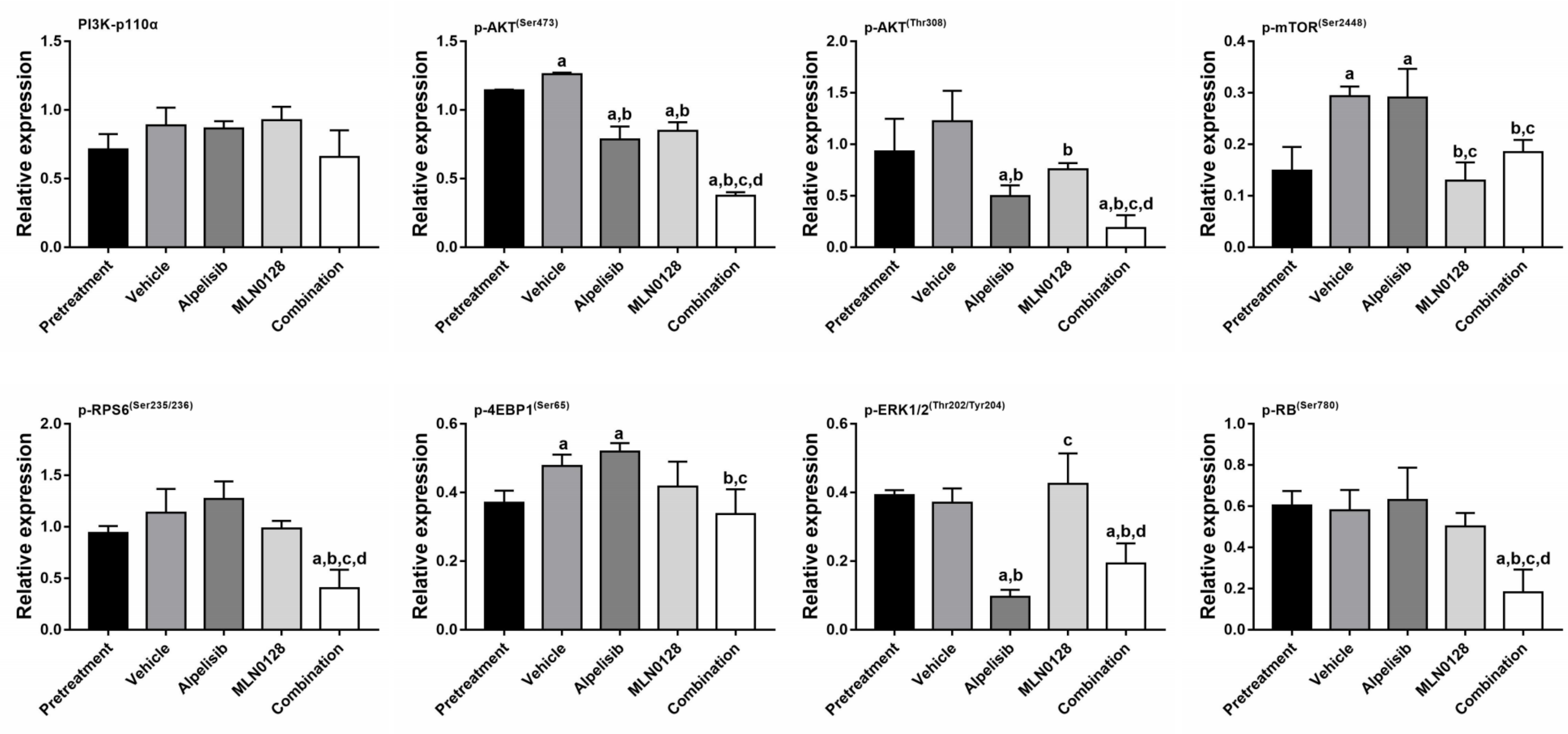


**Supplementary Fig. 28**

Effects of alpelisib/MLN0128 treatment on the levels of putative target proteins in livers from c-Met/H1047R mice. The results of Western blot analysis in Figure 6 were quantified to analyze AKT/mTOR, Ras/MAPK and proliferation pathways in pretreated, treated with vehicle, alpelisib, MLN0128 and alpelisib/MLN0128 c-Met/H1047R mice. Tukey–Kramer test: at least $P < 0.05$. a, vs. Pretreatment; b, vs. Vehicle; c, vs. Alpelisib; d, vs. MLN0128.

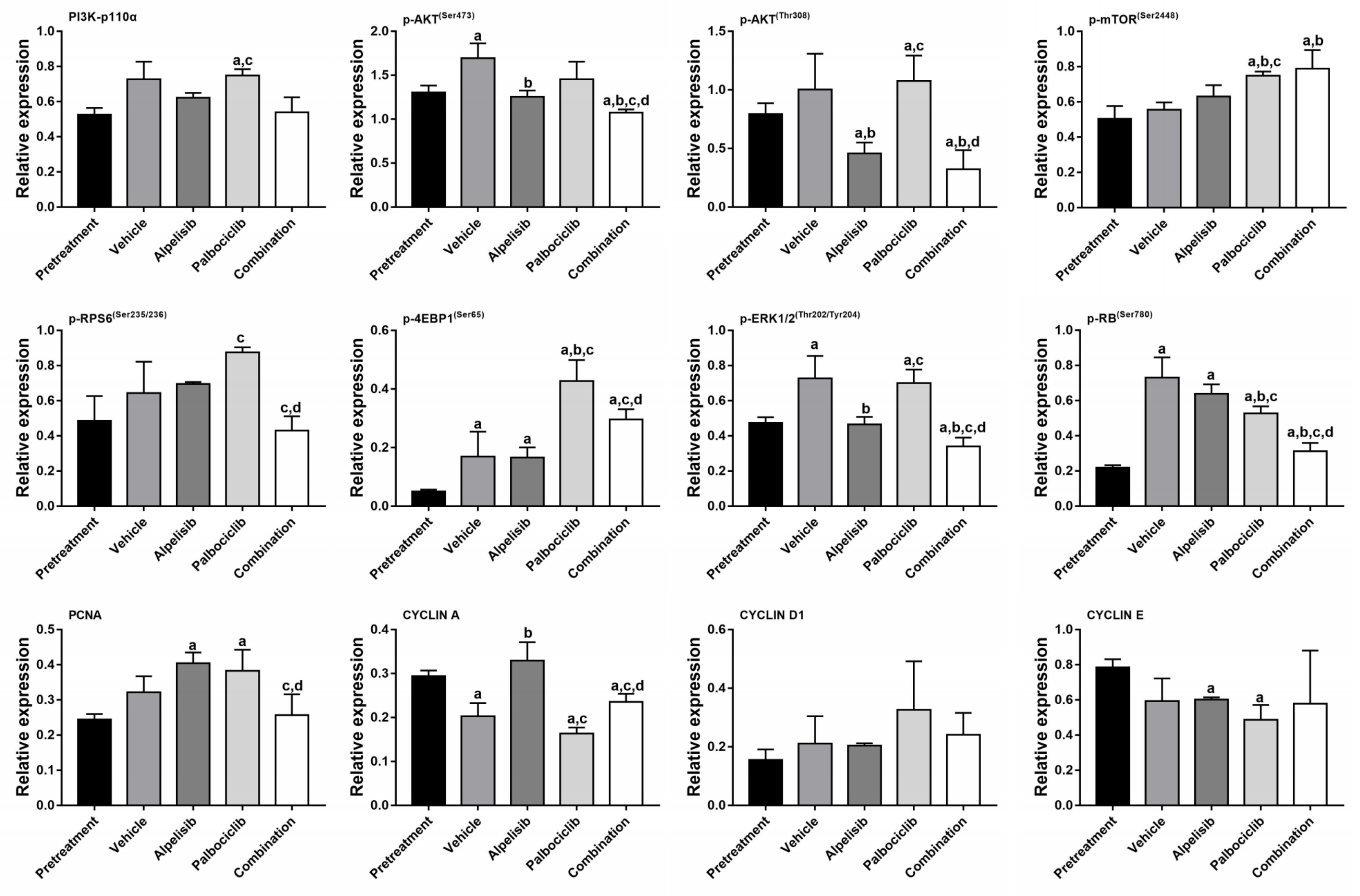

**Supplementary Fig. 29**

Effects of alpelisib/palbociclib treatment on the levels of putative target proteins in livers from c-Met/H1047R mice. The results of Western blot analysis in Figure 8 were quantified to analyze AKT/mTOR, Ras/MAPK, and proliferation pathways in pretreated, treated with vehicle, alpelisib, palbociclib and alpelisib/palbociclib c-Met/H1047R mice. Tukey–Kramer test: at least $P < 0.05$. a, vs. Pretreatment; b, vs. Vehicle; c, vs. Alpelisib; d, vs. Palbociclib.

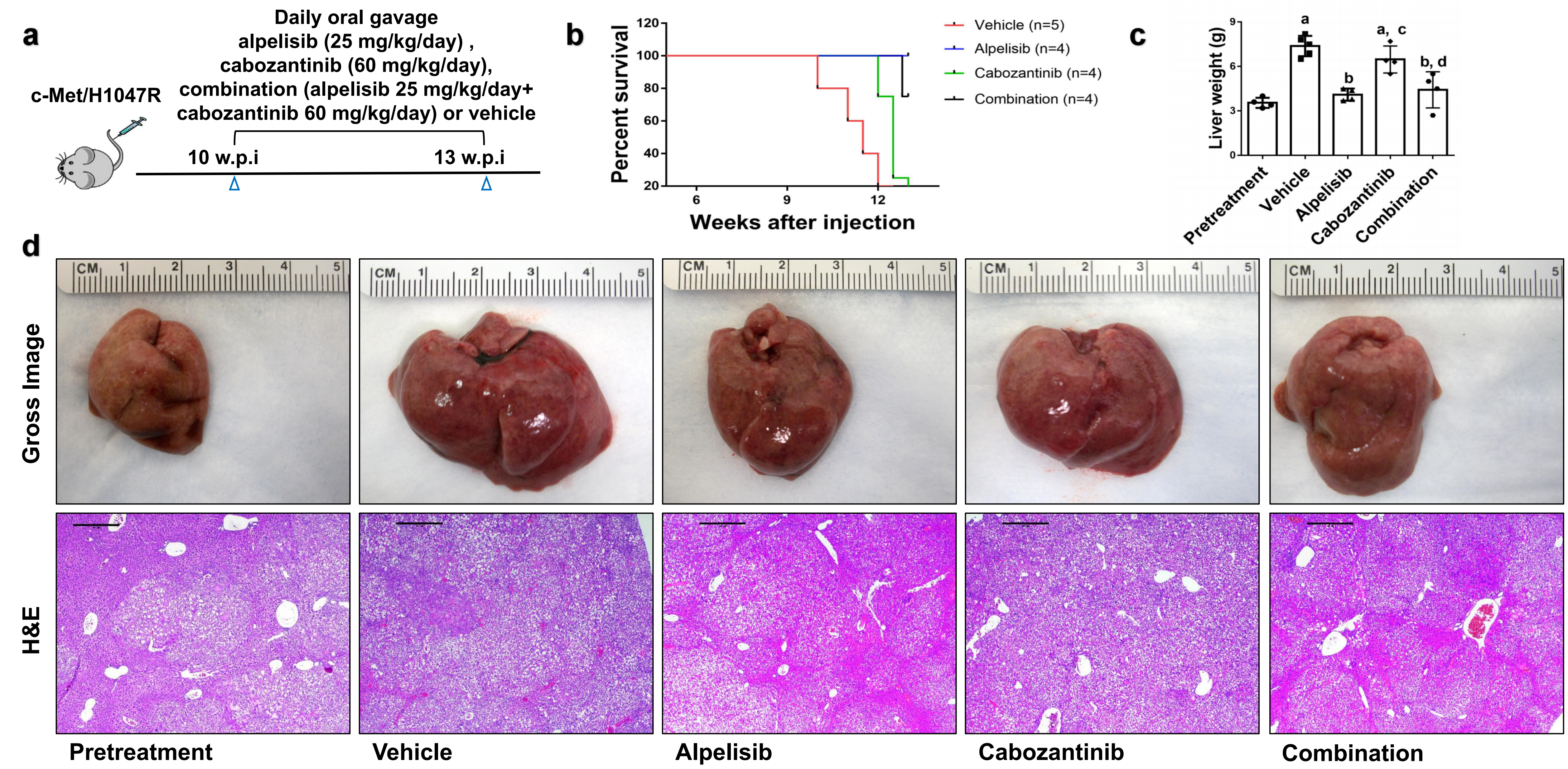


**Supplementary Fig. 30**

Combined alpelisib/cabozantinib has no efficacy in inhibiting HCC progression from c-Met/H1047R mice. **a,** Study design. w.p.i., weeks post-injection. **b,** Survival curve of c-Met/H1047R mice pretreated, treated with vehicle, alpelisib, cabozantinib and alpelisib/cabozantinib. **c,** Liver weight of pretreated, treated with vehicle, alpelisib, cabozantinib and alpelisib/cabozantinib c-Met/H1047R mice. **d,** Gross images and H&E staining of livers from pretreated, treated with vehicle, alpelisib, cabozantinib and alpelisib/cabozantinib c-Met/H1047R mice. Magnification ×40; scale bar =500μm.

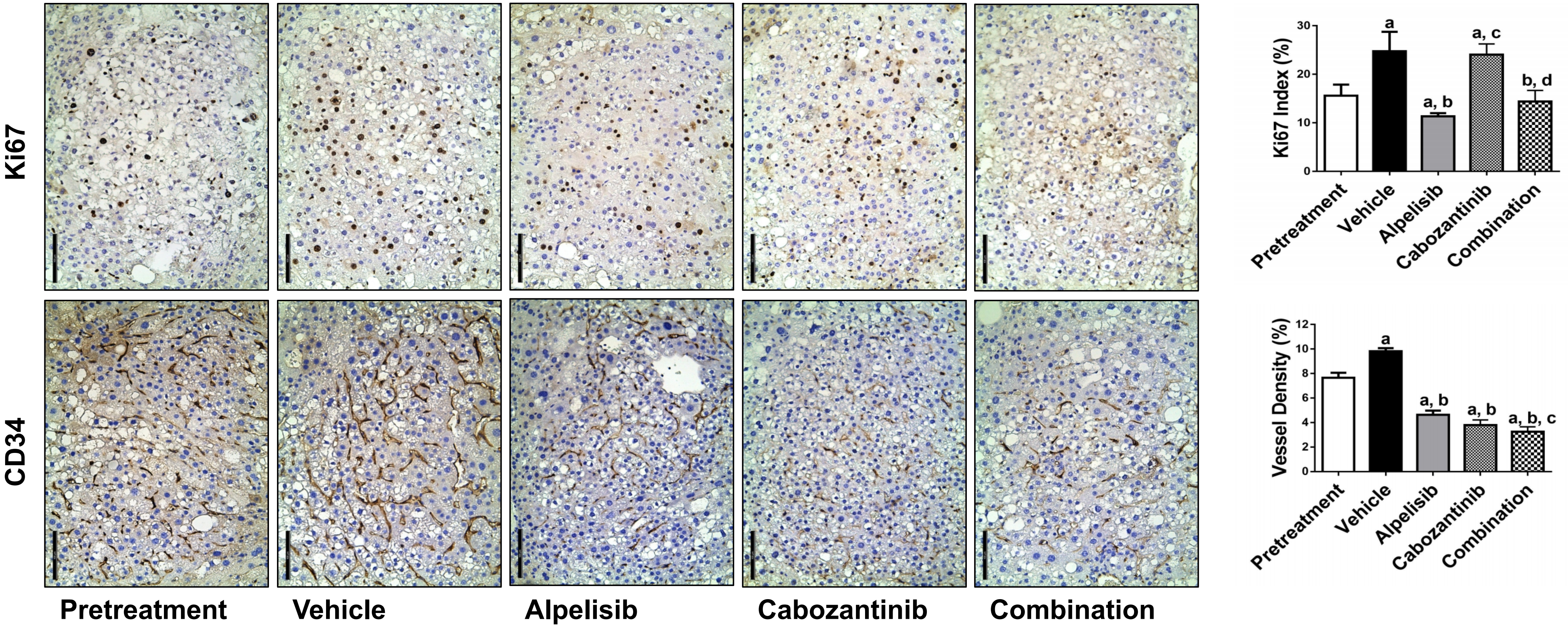


**Supplementary Fig. 31**

Combined alpelisib/cabozantinib has no efficacy in inhibiting HCC progression from c-Met/H1047R mice. Ki67 (magnification ×200; scale bar =100μm) and CD34 (magnification ×200; scale bar =100μm) staining in livers from c-Met/H1047R mice. Ki67-positive cells were counted and quantified as proliferation index. CD34 staining was quantified and represented as the percentage of the positive staining area of the whole section area. Tukey–Kramer test: at least $P < 0.05$. a, vs. Pretreatment; b, vs. Vehicle; c, vs. Alpelisib; d, vs. Cabozantinib.

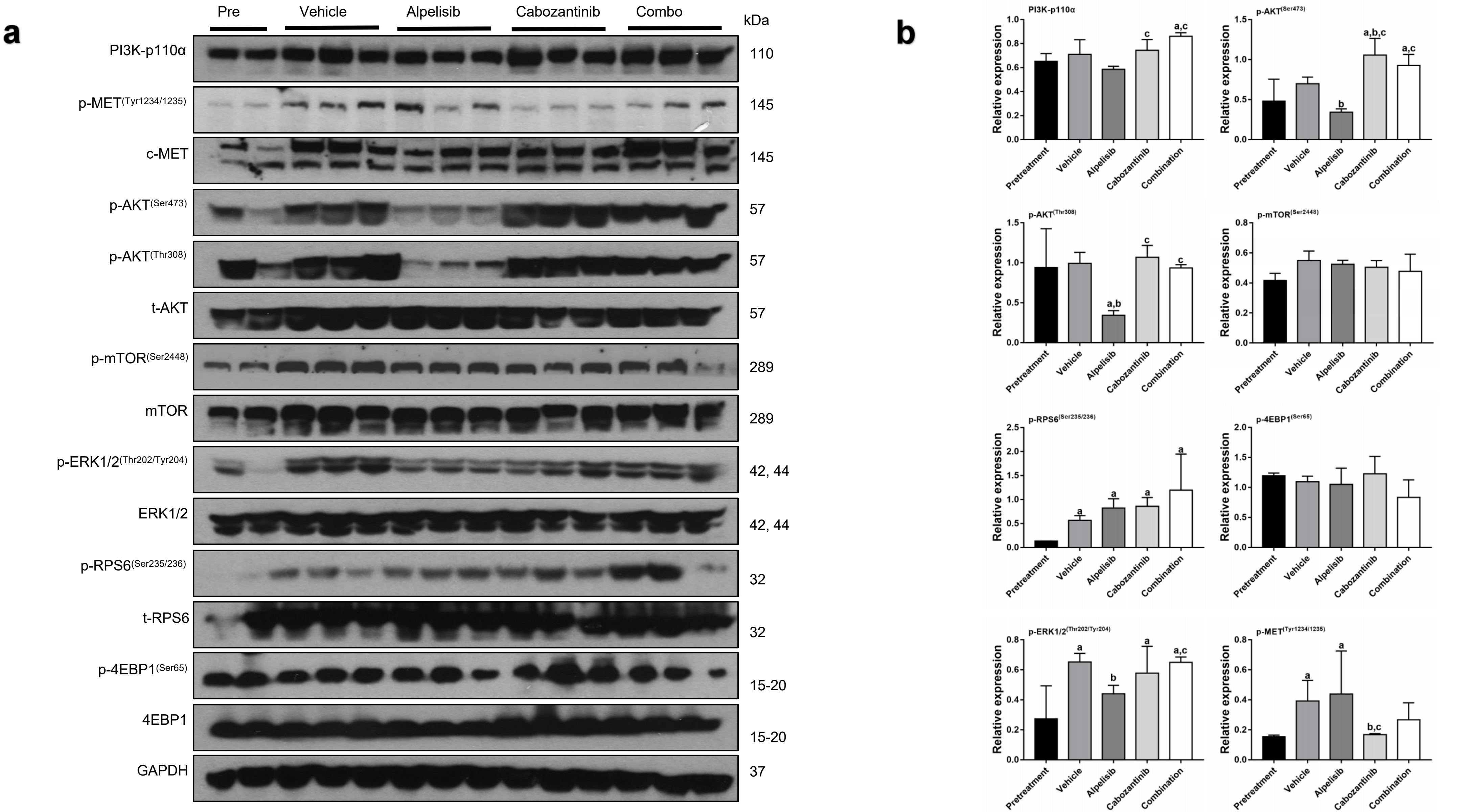


**Supplementary Fig. 32**

Effects of alpelisib/cabozantinib treatment on the levels of putative target proteins in livers from c-Met/H1047R mice. **a,** Western blot analysis of AKT/mTOR and Ras/MAPK pathways in pretreated, treated with vehicle, alpelisib, cabozantinib and alpelisib/cabozantinib c-Met/H1047R mice. **b,** Quantification analysis of AKT/mTOR and Ras/MAPK pathways in pretreated, treated with vehicle, alpelisib, cabozantinib and alpelisib/cabozantinib c-Met/H1047R mice. Tukey–Kramer test: at least $P < 0.05$. a, vs. Pretreatment; b, vs. Vehicle; c, vs. Alpelisib. Abbreviations: Pre, Pretreatment; Combo, combined alpelisib/cabozantinib treatment.

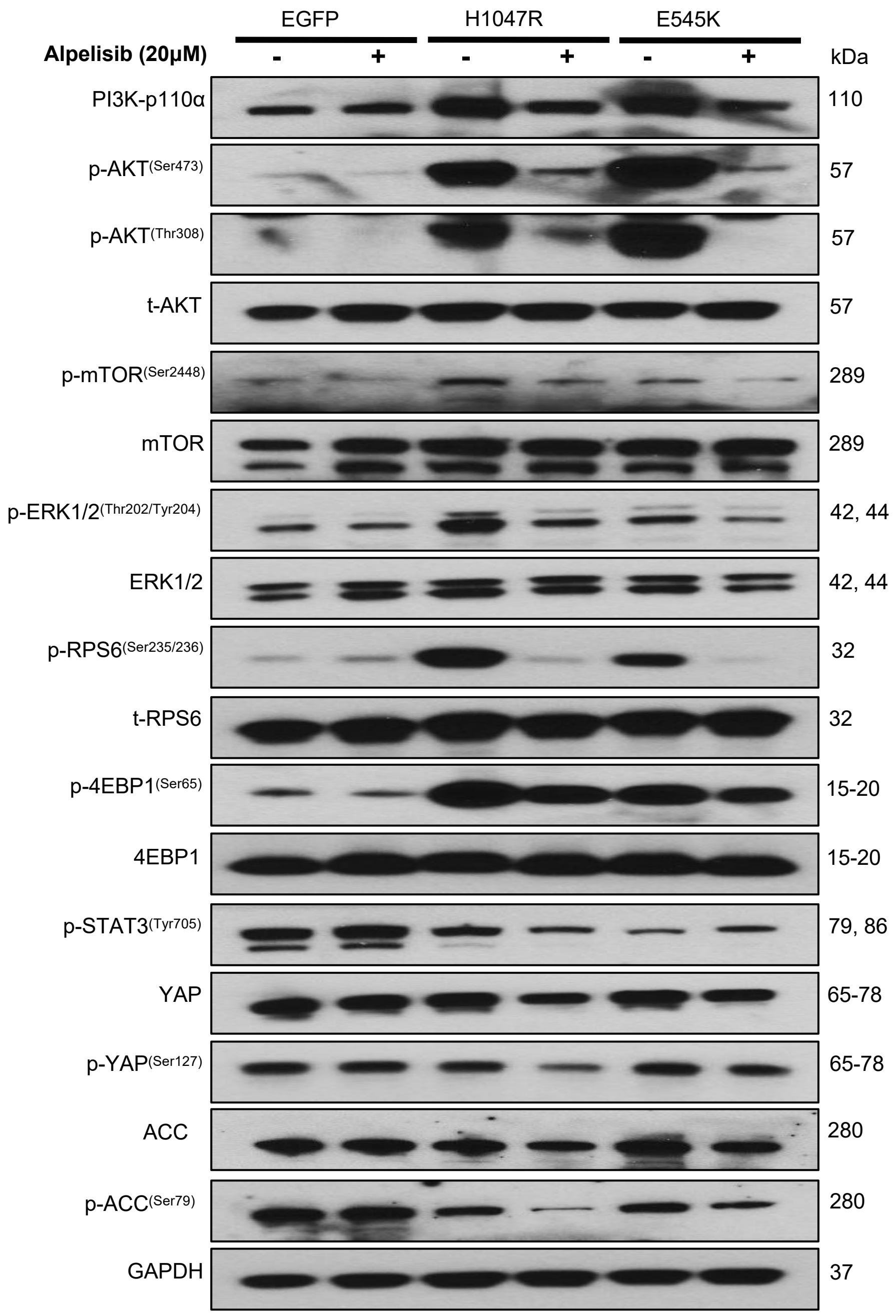


**Supplementary Fig. 33**

Transfection with the *PIK3CA* mutation construct increases the sensitivity to alpelisib treatment of HCC cells. Western blot analysis of AKT/mTOR, Ras/MAPK, STAT3, and YAP pathways after treatment with alpelisib in SNU387 cells.

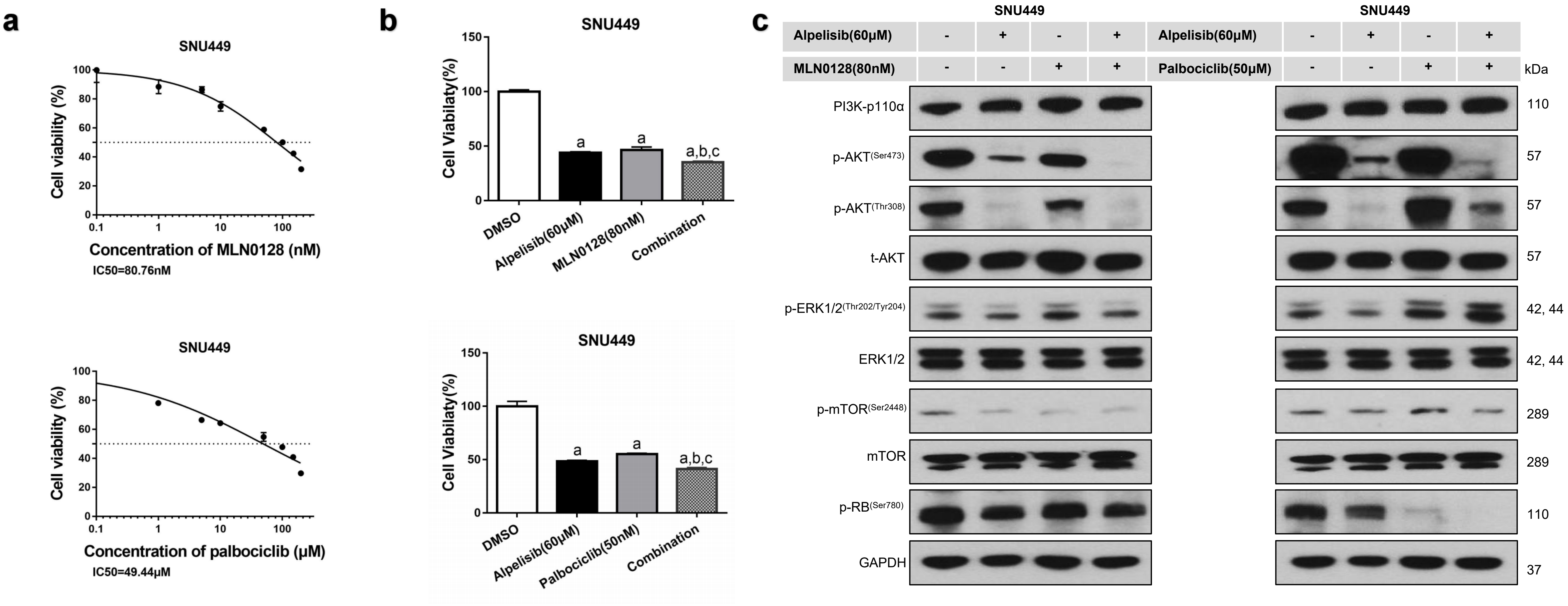


**Supplementary Fig. 34**

Effects of combined alpelisib/MLN0128 or alpelisib/palbociclib treatment on SNU449 wild-type cell lines. SNU449 wild-type cell lines were seeded in 24-well plates at $2.5\times10^5$ and treated for 48 hours. **a,** The $IC_{50}$ values of SNU449 wild-type cell line to the treatment of MLN0128 and palbociclib were calculated. **b,** Combined alpelisib/MLN0128 or alpelisib/palbociclib treatment reduced cell proliferation in SNU449 wild-type cell lines. **c,** Western blot analysis of AKT/mTOR and Ras/MAPK pathways in SNU449 wild-type cell lines. Tukey–Kramer test: at least $P < 0.05$. a, vs. DMSO; b, vs. Alpelisib; c, vs. MLN0128 or Palbociclib.
