# Supplementary material for: Alpelisib combination treatment as novel targeted therapy against hepatocellular carcinoma
Source: Cell Death Dis. 2021 Oct 8;12(10):920. doi: 10.1038/s41419-021-04206-5 (PMC8501067; doi:10.1038/s41419-021-04206-5)
Supplement: Supplementary file 2 — Supplementary Table S1 [file 41419_2021_4206_MOESM2_ESM.docx]

**Supplementary Table 1: List of the antibodies used in the study**

| **Antibody** | **Company** | **Catalog number** | **Dilution** |
| --- | --- | --- | --- |
| Ki67 | Cell Signaling Technology | 12202 | 1:150 |
| CD34 | Abcam | ab81289 | 1:2000 |
| GAPDH | Cell Signaling Technology | 5174 | 1:2000 |
| Pi3k-p110α | Cell Signaling Technology | 4249 | 1:1000 |
| c-Met | Invitrogen | 71-8000 | 1:400 |
| Phospho-Met ^(Tyr1234/1235)^ | Cell Signaling Technology | 3077 | 1:200 |
| Akt | Cell Signaling Technology | 9272 | 1:1000 |
| Phospho-Akt^(S308)^ | Cell Signaling Technology | 13038 | 1:1000 |
| Phospho-Akt^(S473)^ | Cell Signaling Technology | 3787 | 1:1000 |
| mTOR | Cell Signaling Technology | 2983 | 1:1000 |
| Phospho-mTOR^(Ser2448)^ | Cell Signaling Technology | 2971 | 1:1000 |
| Erk1/2 | Cell Signaling Technology | 9102 | 1:1000 |
| Phospho-Erk1/2^(Thr202/Tyr204)^ | Cell Signaling Technology | 4370 | 1:1000 |
| Rps6 | Cell Signaling Technology | 2217 | 1:1000 |
| Phospho-Rps6^(Ser235/236)^ | Cell Signaling Technology | 4858 | 1:1000 |
| 4EBP1 | Cell Signaling Technology | 9644 | 1:1000 |
| Phospho-4EBP1^(Ser65)^ | Cell Signaling Technology | 9451 | 1:1000 |
| Phospho-Rb^(Ser780)^ | Cell Signaling Technology | 9307 | 1:500 |
| PCNA | Cell Signaling Technology | 2586 | 1:1000 |
| Cyclin A | Santa Cruz Biotechnology | sc-751 | 1:500 |
| Cyclin D1 | Abcam | ab134175 | 1:5000 |
| Cyclin E | Santa Cruz Biotechnology | sc-481 | 1:200 |
| P16 | Abcam | ab51243 | 1:200 |
| P21 | BD Pharmingen™ | 556430 | 1:200 |
| P53 | Santa Cruz Biotechnology | sc-126 | 1:200 |
| Survivin | Cell Signaling Technology | 2808 | 1:1000 |
| Cleaved-caspase3 | Cell Signaling Technology | 9664 | 1:1000 |
| HA-Tag | Cell Signaling Technology | 2367 | 1:1000 |
| HIF-1α | Cell Signaling Technology | 36169 | 1:1000 |
| FASN | Cell Signaling Technology | 3180 | 1:1000 |
| ACC | Cell Signaling Technology | 3676 | 1:1000 |
| p-ACC^(Ser79)^ | Cell Signaling Technology | 11818 | 1:1000 |
| SCD1 | Cell Signaling Technology | 2794 | 1:1000 |
| SGK3 | Cell Signaling Technology | 8156 | 1:1000 |
| MCL1 | Cell Signaling Technology | 94296 | 1:1000 |
| EphA2 | Cell Signaling Technology | 6997 | 1:1000 |
| p-Stat3^(Tyr705)^ | Cell Signaling Technology | 9145 | 1:1000 |
| p-Smad2^(Ser465/467)^/3^(Ser423/425)^ | Cell Signaling Technology | 8828 | 1:1000 |
| p-AMPKα^(Thr172)^ | Cell Signaling Technology | 2535 | 1:1000 |
| AMPKα | Cell Signaling Technology | 5831 | 1:1000 |
| β-Actin | Cell Signaling Technology | 4970 | 1:1000 |
| p-Vegfr2^(Tyr1054/1059)^ | Invitrogen | 44-1047G | 1:500 |
| Yap | Cell Signaling Technology | 14074 | 1:1000 |
| p-Yap^(Ser127)^ | Cell Signaling Technology | 13008 | 1:1000 |
